# Supplementary material for: Fibroblast-enriched endoplasmic reticulum protein TXNDC5 promotes pulmonary fibrosis by augmenting TGFβ signaling through TGFBR1 stabilization
Source: Nat Commun. 2020 Aug 26;11:4254. doi: 10.1038/s41467-020-18047-x (PMC7449970; doi:10.1038/s41467-020-18047-x)
Supplement: Supplementary file 1 — Supplementary Information [file 41467_2020_18047_MOESM1_ESM.pdf]

Supplementary Information

Fibroblast-enriched Endoplasmic Reticulum Protein TXNDC5 Promotes Pulmonary Fibrosis by Augmenting TGFβ Signaling through TGFBR1 Stabilization

Lee et al.

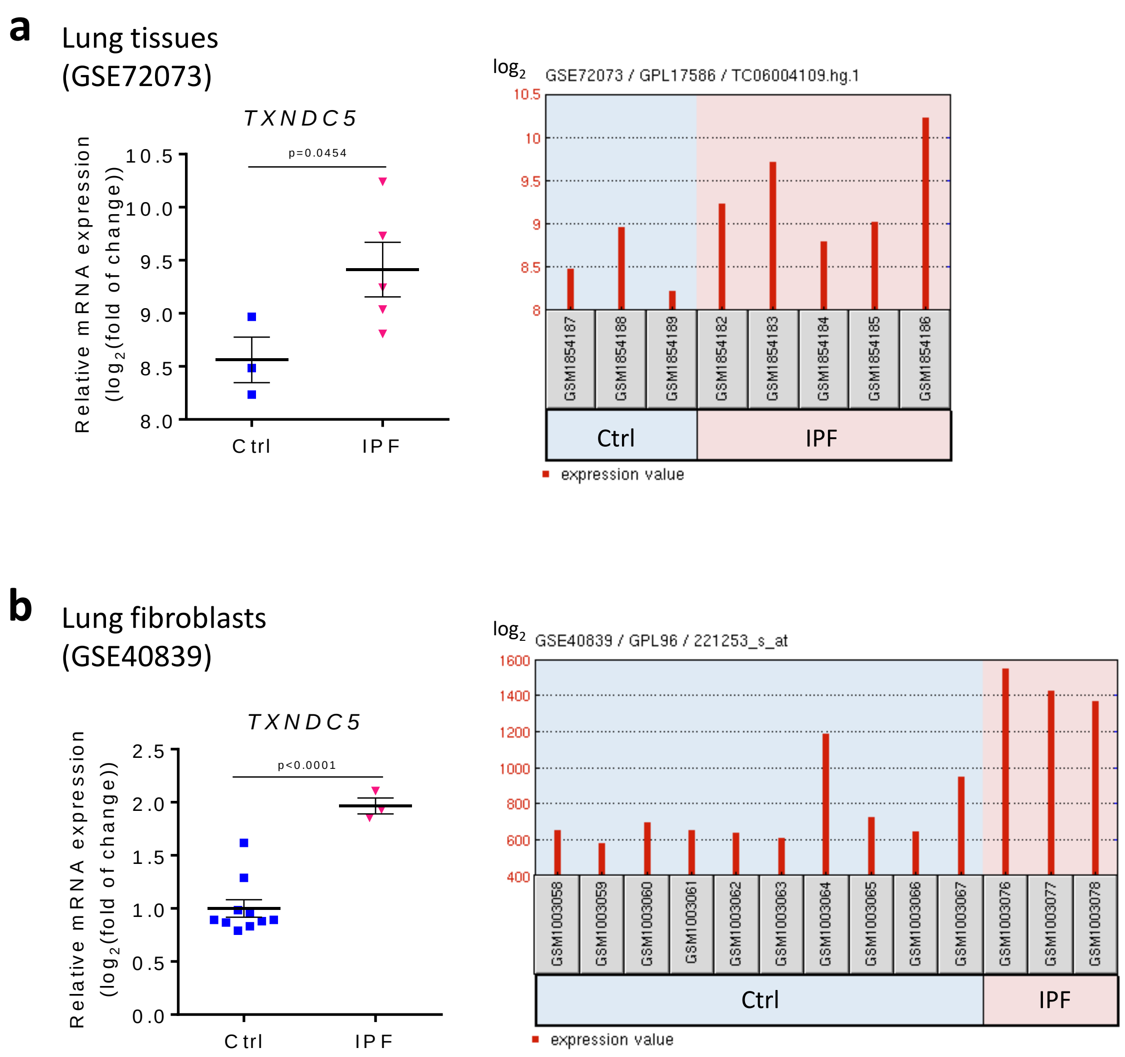

Supplementary Fig. 1 *TXNDC5* transcript was upregulated in human IPF lungs/lung fibroblasts. Re-analyses of microarray data obtained from human IPF lungs (GSE72073) and lung fibroblasts (GSE40839)[1] showed that *TXNDC5* mRNA was significantly upregulated in the lung tissues (Ctrl n=3, IPF n=5 biologically independent samples) **(a)** and lung fibroblasts (Ctrl n=10, IPF n=3 biologically independent samples) **(b)** (Data are presented as mean ± SEM, *P* value determined using two-tailed unpaired *t* test. Source data are provided as a Source Data file. Ctrl: control).

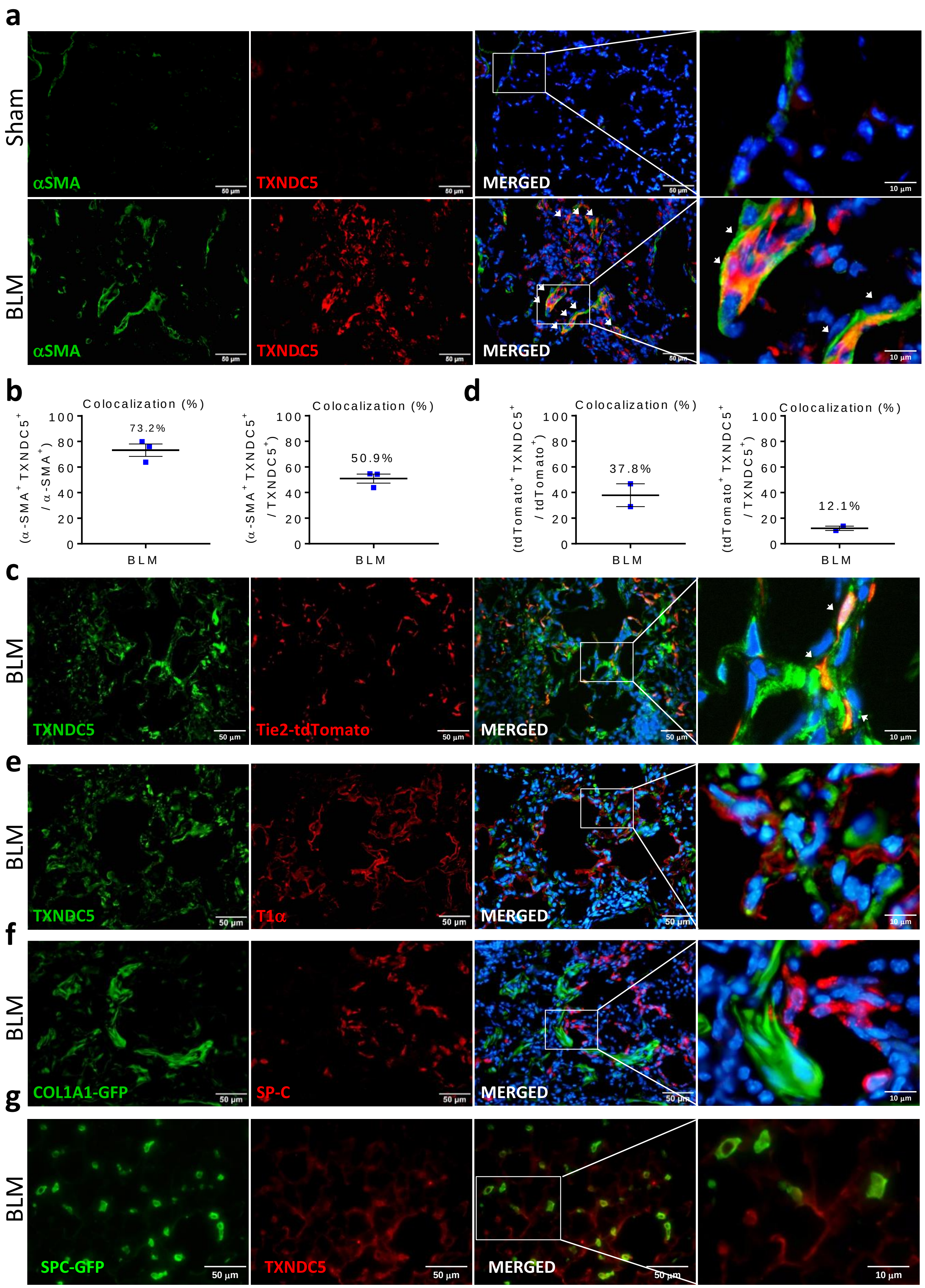

Supplementary Fig. 2 TXNDC5 was co-localized with active myofibroblasts in the mouse lungs. (a-b) IF staining of TXNDC5 (red) and αSMA (green) using lung sections from BLM- and PBS- (Sham)

treated WT mice on day 21 showed a high degree (73%) of co-localization of TXNDC5 with αSMA-positive, activated myofibroblasts in the mouse lungs (n=3 biologically independent animals). **(c-d)** TXNDC5 was only present in ~37% of tdTomato-posive endothelial cells after BLM treatment on day 21(n=2 biologically independent animals). **(e)** T1α (red), a marker for type I pneumocytes, did not co-localize with TXNDC5 (green) in lung sections from BLM-treated WT mice on day 21. **(f)** SP-C (red), a marker for type II pneumocytes, was not present in COL1A1-GFP positive cells from *Col1a1-GFP*^{Tg} mice 21 days following BLM treatment. (g) IF staining of TXNDC5 (red) using lung sections from BLM-treated *SPC-mTmG* mice on day 21 showed that TXNDC5 was rarely expressed in type II pneumocytes, which were marked with GFP (Data are presented as mean ± SEM. Source data are provided as a Source Data file. BLM: bleomycin).

a

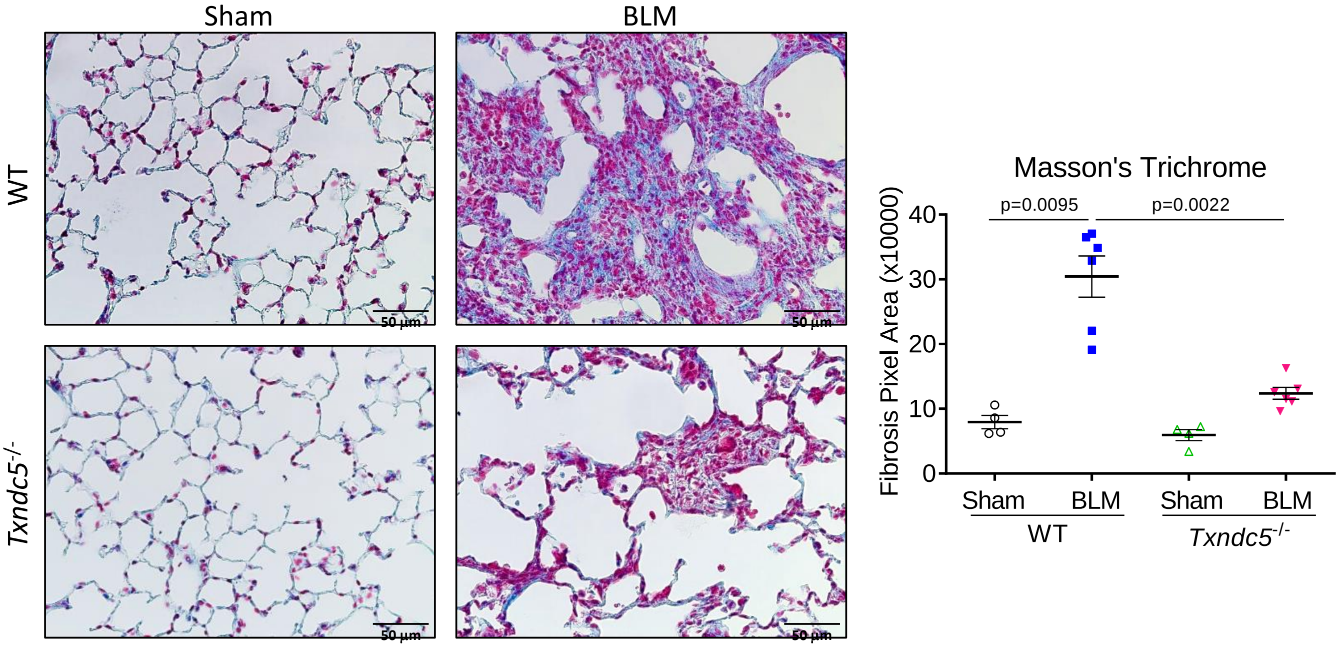

Supplementary Fig. 3 Loss of *Txndc5* ameliorates BLM-induced pulmonary fibrosis.
Representative Masson's trichrome staining (left) and quantification of fibrotic areas (right) of lung sections from WT and *Txndc5*$^{-/-}$ mice 21 days after intra-tracheal BLM or PBS (Sham) instillation (sham n=4, BLM n=6 biologically independent animals per group). BLM-induced lung fibrosis was significantly attenuated in *Txndc5*$^{-/-}$ mice (Data are presented as mean ± SEM, *P* value determined using two-tailed Mann-Whitney *U* test. Source data are provided as a Source Data file. BLM: bleomycin).

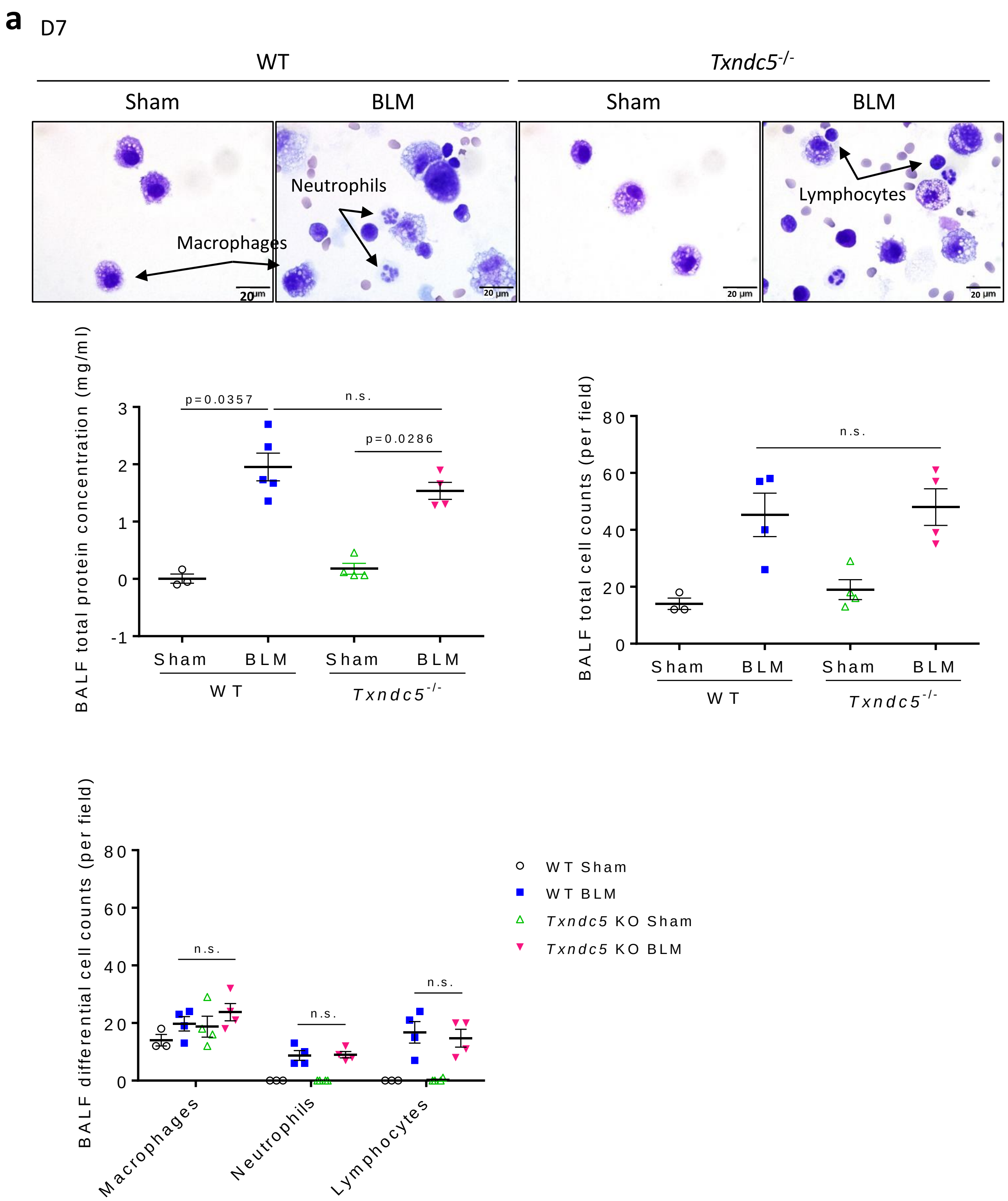

Supplementary Fig. 4 Inflammatory responses in WT and *Txndc5$^{-/-}$* mouse lungs.

(a) Representative images of modified Giemsa staining of the BALF from WT and *Txndc5$^{-/-}$* mice 7 days following sham procedure or BLM treatment (top panel). The total protein content (WT sham n=3, WT BLM n=5, *Txndc5$^{-/-}$* sham n=4, *Txndc5$^{-/-}$* BLM n=4 biologically independent animals) and number of inflammatory cell (macrophage, neutrophil and lymphocytes) infiltration (WT sham n=3, WT BLM n=4, *Txndc5$^{-/-}$* sham n=4, *Txndc5$^{-/-}$* BLM n=4 biologically independent animals) were quantified in each group (bottom panel) (Data are presented as mean ± SEM, *P* value determined using two-tailed Mann-Whitney *U* test. Source data are provided as a Source Data file. n.s.: non-significant BLM: bleomycin, BALF:

bronchoalveolar lavage fluid).

a

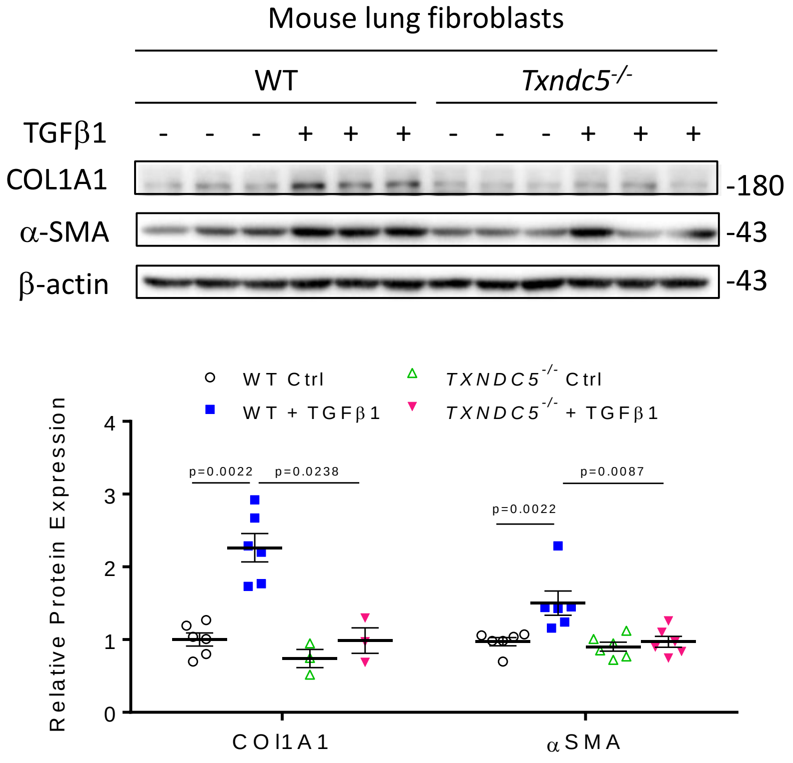

b

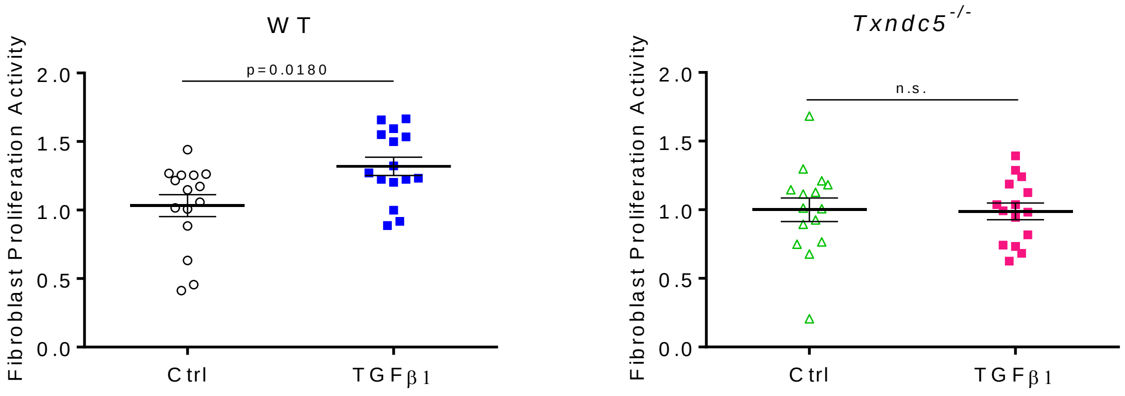

Supplementary Fig. 5 TXNDC5 is required for TGFβ1-induced MLF activation and proliferation.(a) Immunoblot analyses of COL1A1 and αSMA using protein lysates of primary MLF isolated from WT and *Txndc5*$^{-/-}$ mice, with and without TGFβ1 (10 ng/ml) treatment (COL1A1, WT n=6, *Txndc5*$^{-/-}$ n=3 biologically independent animals; αSMA n=6 biologically independent animals per group). TGFβ1 treatment induced marked upregulation of COL1A1 and αSMA in WT MLF, which was completely abolished in *Txndc5*$^{-/-}$ MLF. **(b)** TGFβ1 treatment significantly increased the proliferation activity of WT, but not *Txndc5*$^{-/-}$, MLF (n=15 biologically independent samples per group) (Data are presented as mean ± SEM, *P* value determined using two-tailed Mann-Whitney *U* test. Source data are provided as a Source Data file. n.s.: non-significant, Ctrl: control).

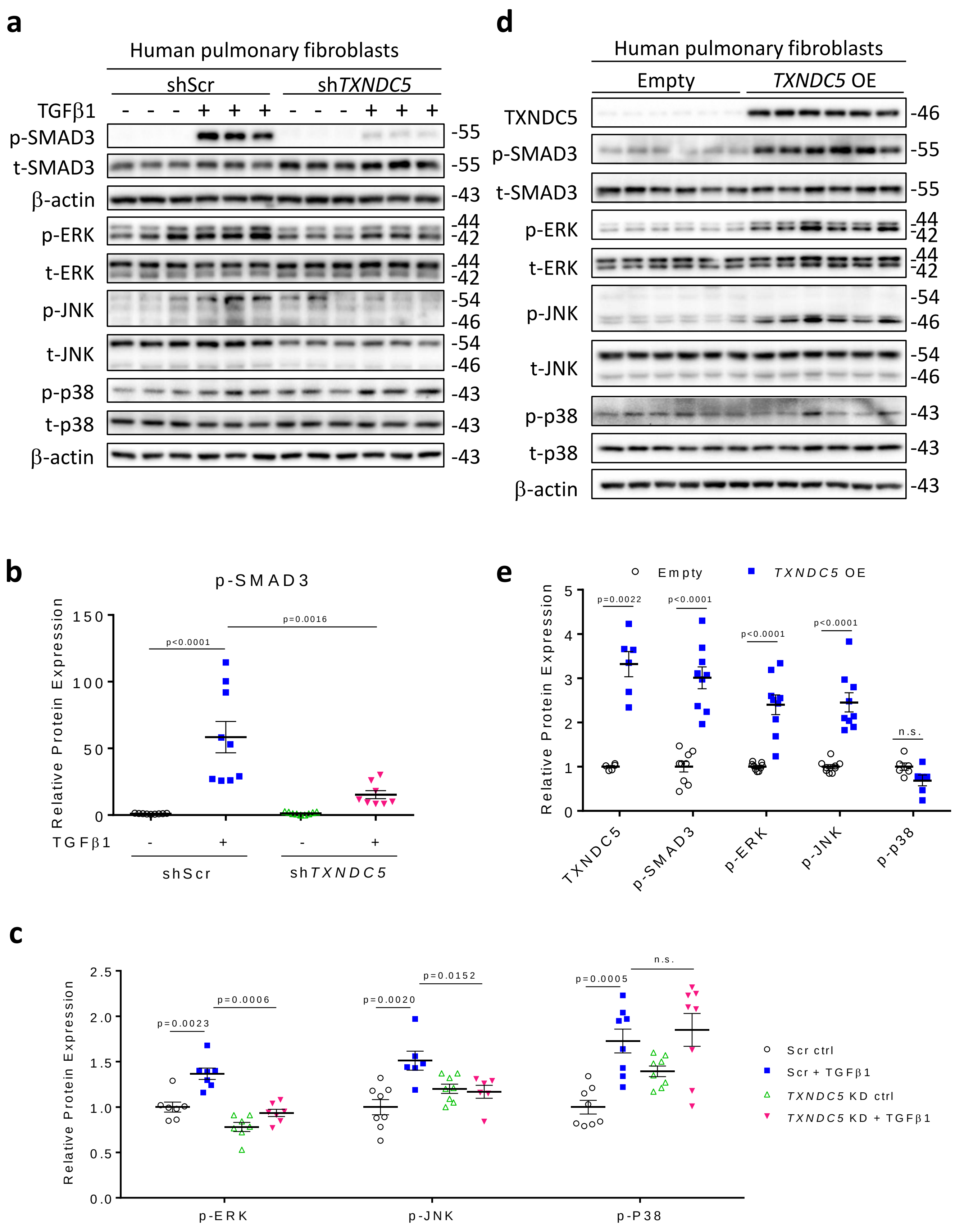

Supplementary Fig. 6 TXNDC5 triggers canonical and non-canonical TGFβ1 signaling pathway.

(a-c) TGFβ1 treatment in control (shScr) HPF led to marked increases in the phosphorylation of canonical (SMAD3) (n=9,9,9,8 biologically independent samples) and non-canonical (JNK, ERK and p38) (p-ERK n=7, p-JNK n=8,6,8,6, p-p38 n=8 biologically independent samples) signaling molecules downstream of

TGFβ pathway. *TXNDC5* depletion (sh*TXNDC5*) significantly reduced the phosphorylation of SMAD3, JNK and ERK, but not p38, in HPF following TGFβ1 treatment. **(d and e)** Overexpression of *TXNDC5* in HPF resulted in significantly increased phosphorylation of SMAD3, JNK and ERK, without affecting p38 phosphorylation (TXNDC5 and p-p38 n=6, p-SMAD3, p-ERK and p-JNK n=9 biologically independent samples) (Data are presented as mean ± SEM, *P* value determined using two-tailed Mann-Whitney *U* test. Source data are provided as a Source Data file. n.s.: non-significant, shTXNDC5: *TXNDC5* knockdown with shRNA, KD: knockdown, OE: overexpress).

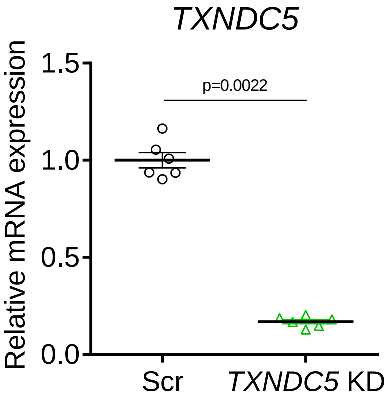

Supplementary Fig. 7 *TXNDC5* knockdown efficiency in HPF.

Knockdown efficiency of lentiviral vectors carrying *TXNDC5*-targeted shRNA in HPF (n=6 biologically independent samples per group) (Data are presented as mean ± SEM, *P* value determined using two-tailed Mann-Whitney *U* test. Source data are provided as a Source Data file. Scr: scramble, KD: knockdown).

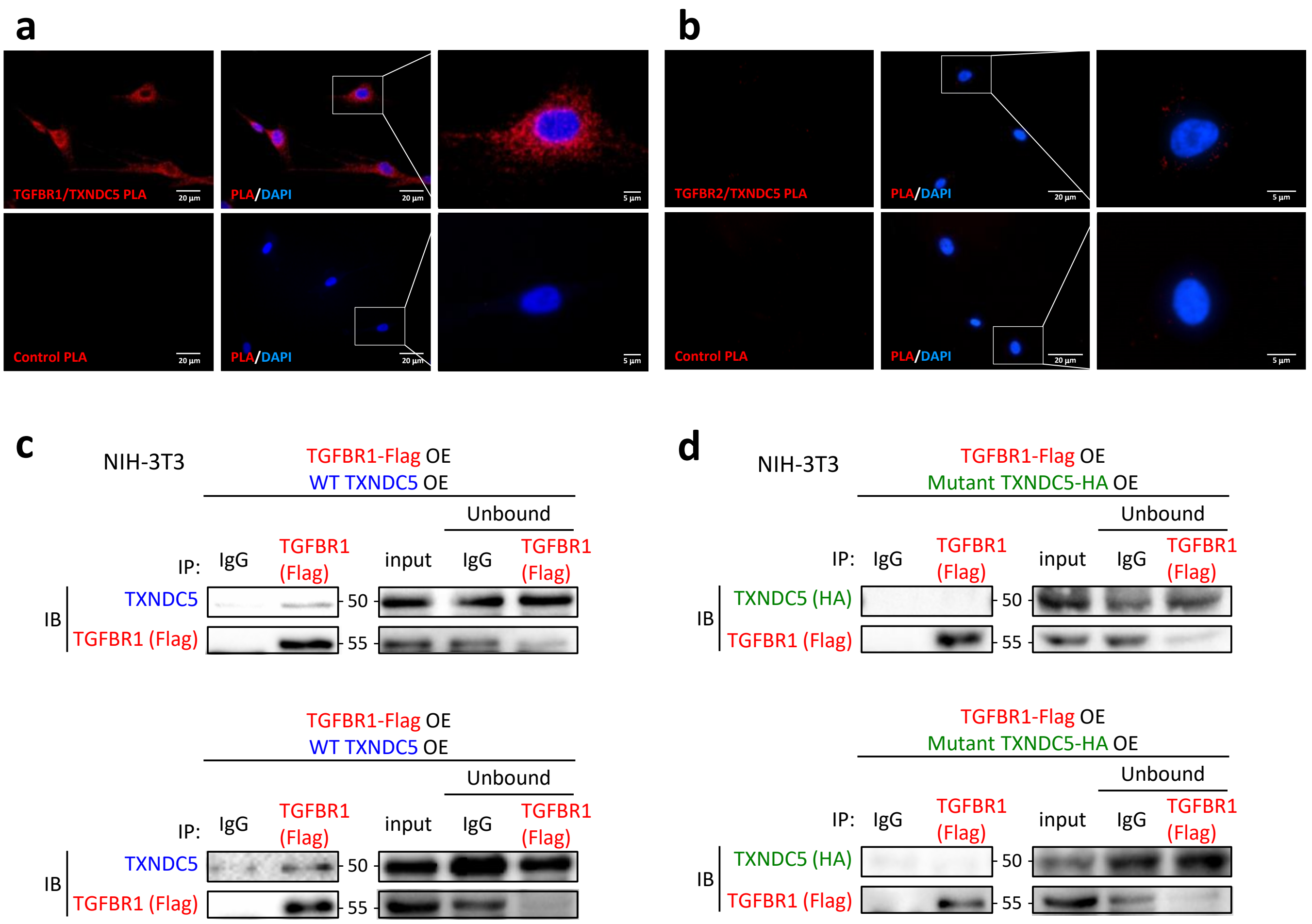

Supplementary Fig. 8 TXNDC5 interacted with TGFBR1.

(a) An *in situ* proximity ligation assay (PLA) showed physical interaction between TXNDC5 and TGFBR1 in the perinuclear ER (red) in HPF (inset). **(b)** PLA did not reveal measurable interaction between TXNDC5 and TGFBR2. **(c)** Protein co-immunoprecipitation experiments were performed in NIH-3T3 mouse fibroblasts co-transfected with TGFBR1-Flag and either WT TXNDC5-HA or **(d)** AAA-mutant TXNDC5-HA mRNAs (scheme of WT and mutant TXNDC5-AAA proteins are shown in the bottom panel). These experiments revealed strong physical interaction between TGFBR1 and WT, but not with AAA-mutant, TXNDC5 in fibroblasts. Two sets of representative blots were shown for each condition. Source data are provided as a Source Data file. OE: overexpress

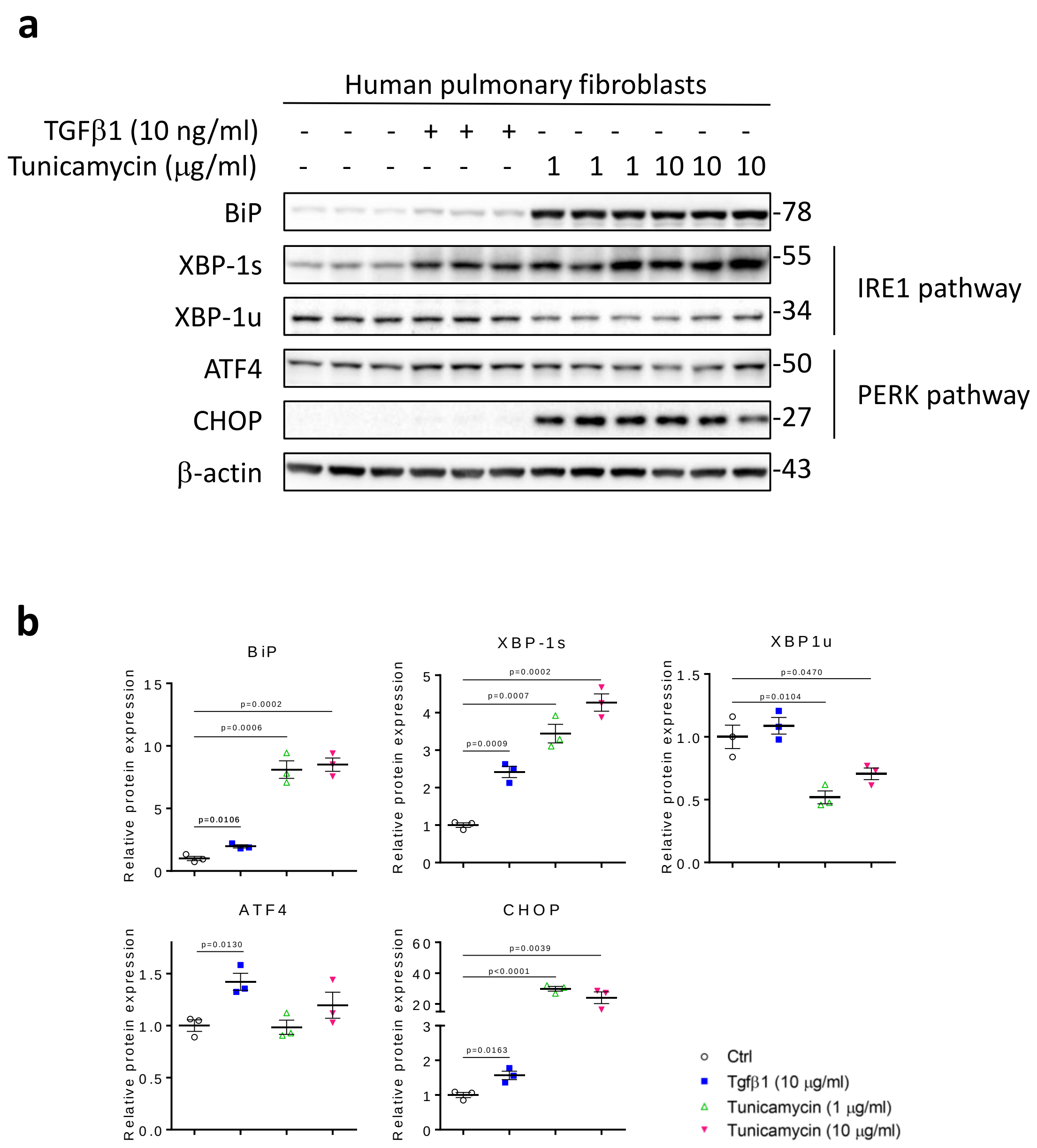

Supplementary Fig. 9 TGFβ1 treatment triggered increased ER stress in HPF.

Immunoblots **(a)** and quantification **(b)** showing that TGFβ1 treatment for 48h induced marked increases in the expression levels of protein components of ER stress pathways, including BiP, XBP-1s, ATF4 and CHOP. Tunicamycin, a potent ER stress inducer, was used as positive control. (XBP-1 exists in the transcriptionally inactive unspliced form XBP-1u and the active spliced form XBP-1s (n=3 biologically independent samples per group). Data are presented as mean ± SEM, *P* value determined using two-tailed unpaired *t* test. Source data are provided as a Source Data file. Ctrl: control).

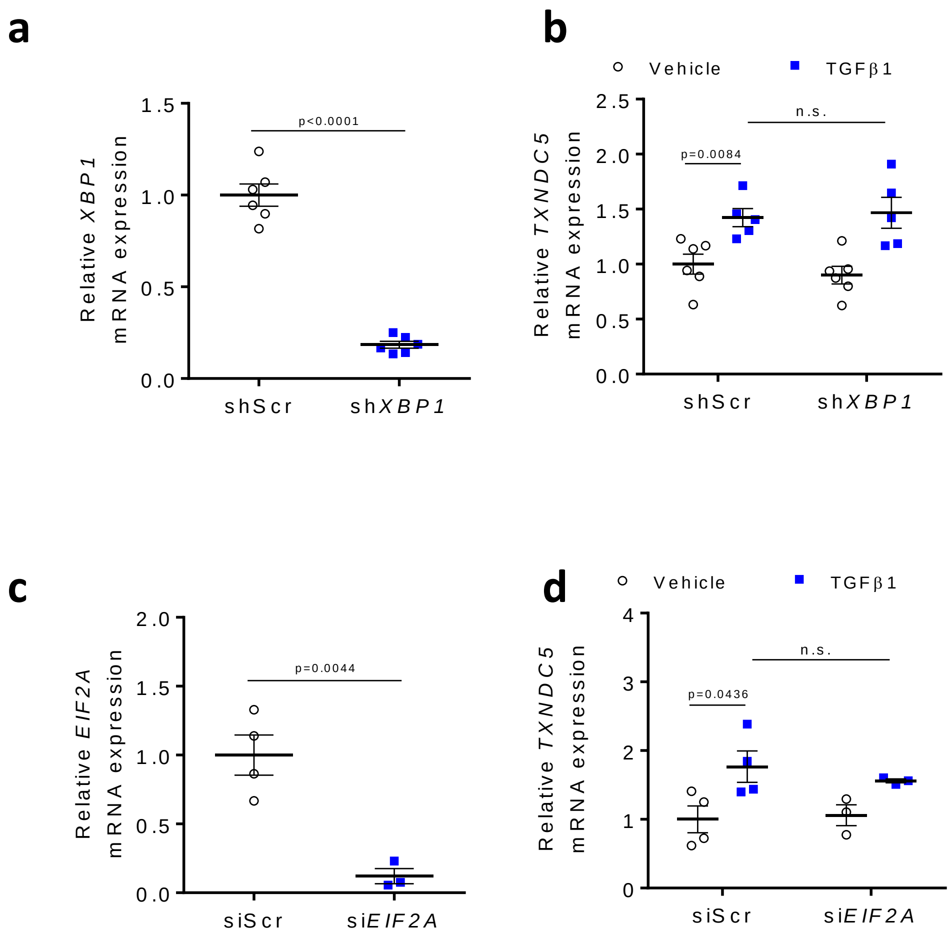

Supplemental Fig. 10 TGFβ1-induced *TXNDC5* upregulation was independent of XBP1 and EIF2A. **(a)** *XBP1* (IRE1 pathway) was knocked down effectively in HPF (n=6 biologically independent samples per group). **(b)** TGFβ1-induced upregulation of TXNDC5 transcript was not affected by knocking down of *XBP1*(Vehicle n=6, TGFβ1 n=5 biologically independent samples). **(c)** The knockdown efficiency of *EIF2A* (PERK pathway) by siRNA in HPF (siScr n=4, siEIF2A n=3 biologically independent samples). **(d)** TGFβ1-induced *TXNDC5* upregulation was independent of *EIF2A* knockdown (siScr n=4, siEIF2A n=3 biologically independent samples per group). (Data are presented as mean ± SEM, *P* value determined using two-tailed unpaired *t* test. Source data are provided as a Source Data file. n.s.: non-significant, shXBP1: *XBP1* knockdown with shRNA, siEIF2A: *EIF2A* knockdown with siRNA)

a

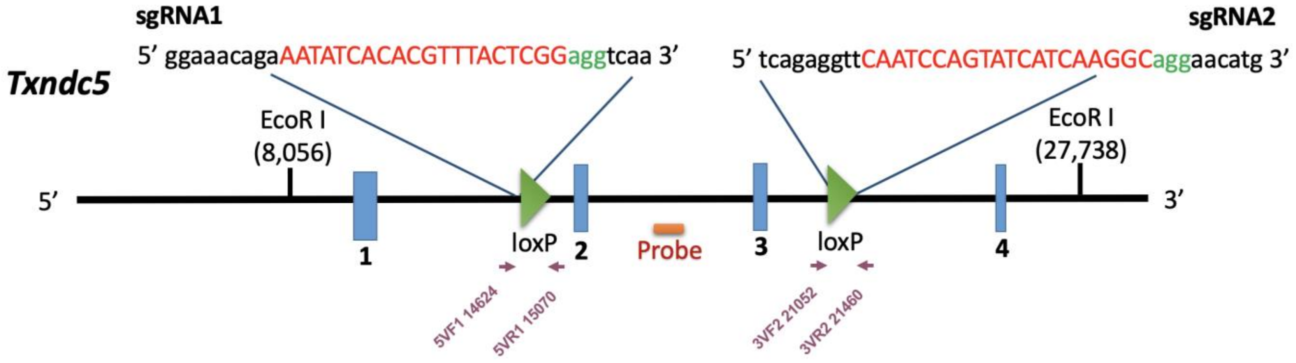

Txndc5 **5' loxP ssODN**

TGAGCTCCAGGGGCACAACAAGCTATACGTTCCAGGAAACAGAAATATCACACGTTTACT
GAATTCATAACTTCGTATAATGTATGCTATACGAAGTTATCGGAGGTCAATTAAAAGTC
TAGGAGGCAGGGTCTGGCATAGAGGTCAAAAAAGAATACTA

Txndc5 **3' loxP ssODN**

GAGGATAACATTTAATTGGGGCTGGTGTATAGGTTCAGAGGTTCAATCCAGTATCATCAA
GAATTCATAACTTCGTATAATGTATGCTATACGAAGTTATGGCAGGAACATGGCAGC
ATCCAGGCAGGCATGGTGCAGAAGGAGCTGAGAGTTCTGTATC

b

(1) Txndc5 5VF1 14624 5'- GGAGGAAGTGATGCCAAACTAGA-3'
(2) Txndc5 5VR1 15070 5'- GTTGTACTTGTCTCCCAGGTCAT-3'
(3) Txndc5 3VF2 21052 5'- GTAGCATAGCCACTATGTCACCA-3'
(4) Txndc5 3VR2 21460 5'- GGATGAGTAATGGAGTCGTGTGT-3'

Primer	PCR product	Note
**5VF1+5VR1**	**447 bp (WT)**<br>**487 bp (Target)**	**5'LoxP site**
**3VF2+3VR2**	**409 bp (WT)**<br>**449 bp (Target)**	**3'LoxP site**

Supplementary Fig. 11 Design of CRISPR/Cas9-mediated creation of floxed TXNDC5 allele.

(a) Schematic design of the Cas9/sgRNA targeting sequences, and two single-strand donor oligonucleotides (ssODN) carrying lox-P knock-in HDR templates flanking exon 2 and exon 3. The sequence of the ssODNs carrying 70 bp homologies on both sides flanking the targeting sites were shown below. Southern blot probes to be used were shown as the red bar. **(b)** Primer sequences and PCR product sizes for genotyping of the *Txndc5*$^{fl/fl}$ mice.

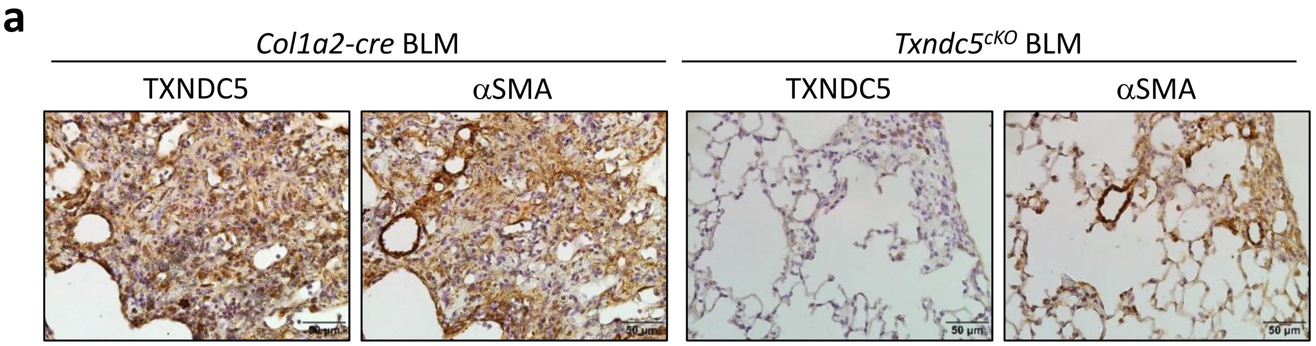

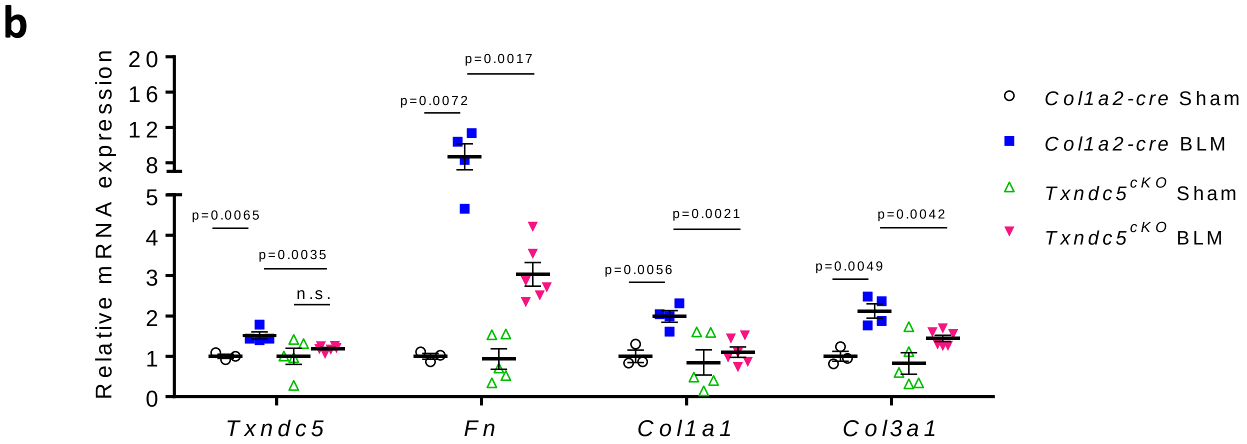

Supplementary Fig. 12 Fibroblasts-specific deletion of *Txndc5* attenuated the extent of PF.

(a) IHC staining on the serial sections of mouse lungs showed a strong expression of TXNDC5 in the αSMA-positive fibrotic foci in the lung tissues from BLM-treated *Col1a2-cre*, but not in *Txndc5*^{cKO}, mice on day 21. **(b)** Fibrogenic protein genes including *Fn, Col1a1* and *Col3a1* were markedly increased in the lung tissues from *Col1a2-cre,* but not in *Txndc5*^{cKO}*,* mice 21 days following BLM treatment (*Col1a2-cre* sham n=3, *Col1a2-cre* BLM n=4, *Txndc5*^{cKO} sham n=5, *Txndc5*^{cKO} BLM n=6 biologically independent animals) (Data are presented as mean ± SEM, *P* value determined using two-tailed unpaired *t* test. Source data are provided as a Source Data file. n.s.: non-significant BLM: bleomycin, *Txndc5*^{cKO}: fibroblasts-specific deletion of *Txndc5*).

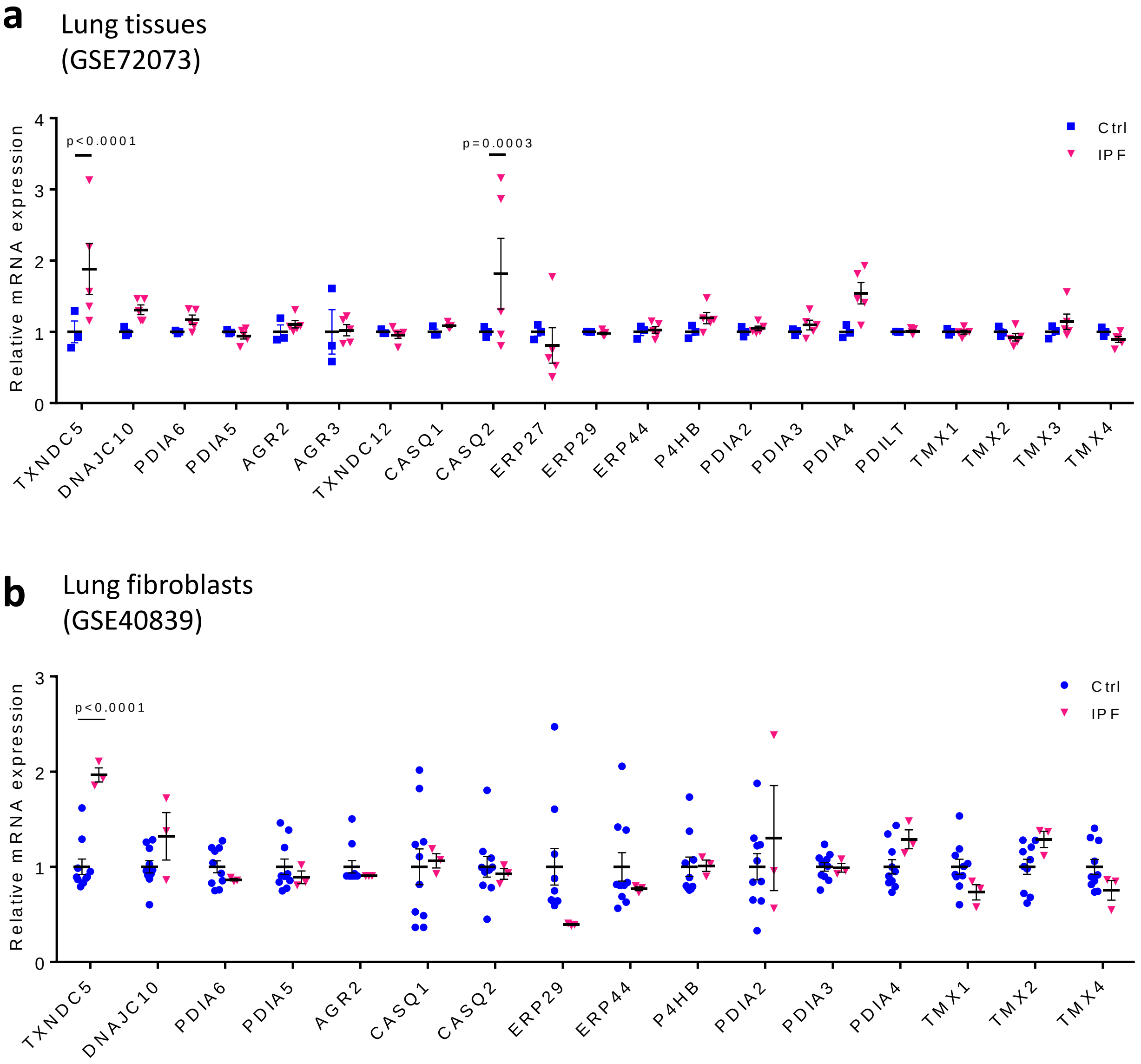

Supplementary Fig. 13 *Txndc5* was the only PDI family gene upregulated in the lung fibroblasts. Re-analysis of microarray data obtained from human IPF lungs (GSE72073, Ctrl n=3, IPF n=5 biologically independent samples) and lung fibroblasts (GSE40839, Ctrl n=10, IPF n=3 biologically independent samples)[1] revealed that *TXNDC5* and *CASQ1* were both upregulated in fibrotic lung tissues **(a)**, while *Txndc5* was the only PDI that was highly upregulated in lung fibroblasts from IPF, compared to those from control, tissues **(b)** (Data are presented as mean ± SEM, *P* value determined using two-tailed Holm-Sidak multiple *t* test. Source data are provided as a Source Data file).

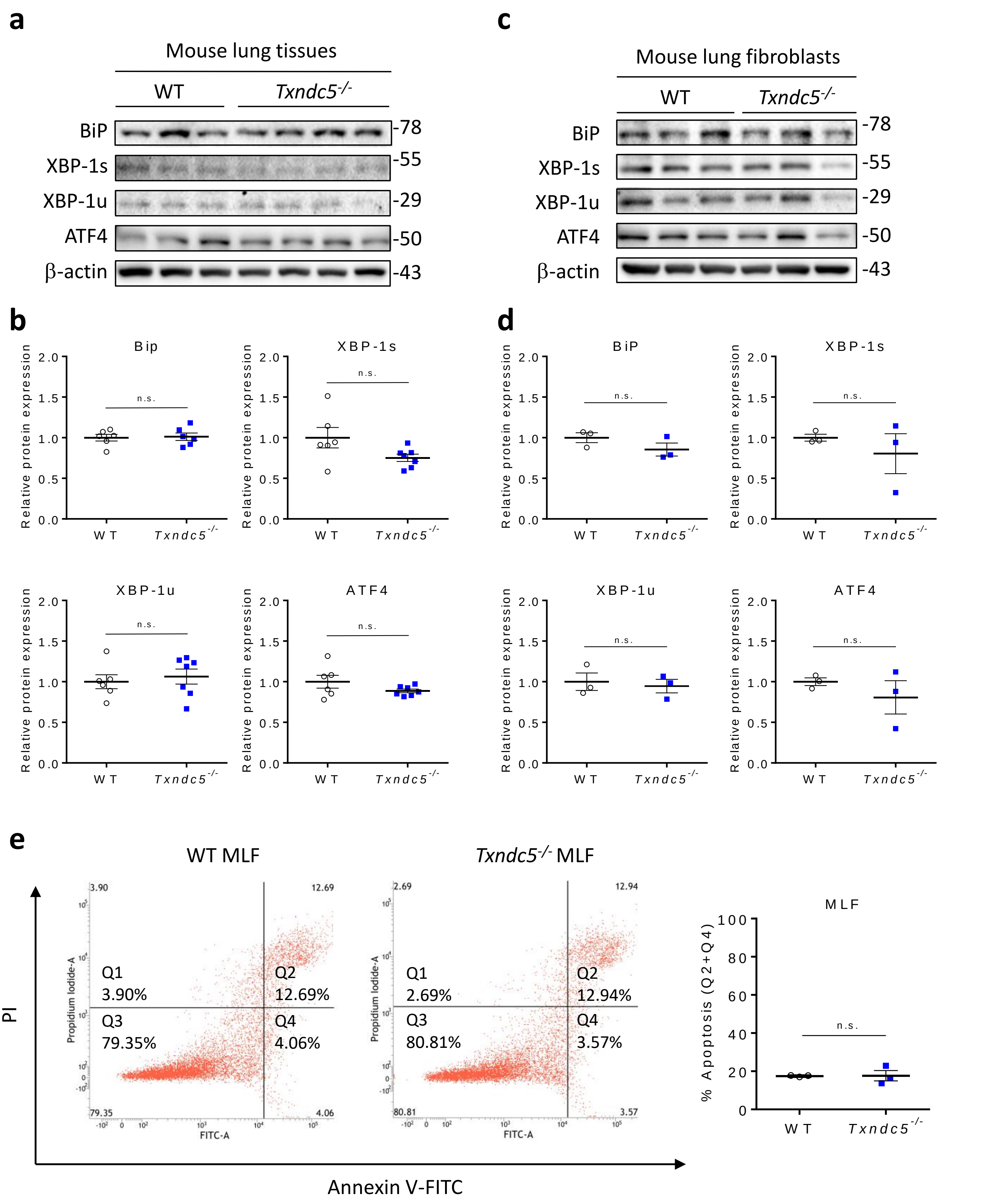

Supplementary Fig. 14 Deletion of *Txndc5* did not lead to increased ER stress or apoptosis. Immunoblots showing that the expression levels of ER stress markers including BiP, XBP-1s, and ATF4 were indistinguishable in WT and *Txndc5*$^{-/-}$ mouse lung tissues (n=6 biologically independent animals per group) (**a and b**) and mouse lung fibroblasts (n=3 biologically independent animals per group) (**c and d**). **(e)** Flow cytometry was conducted on isolated WT and *Txndc5*$^{-/-}$ mouse lung fibroblasts stained with annexin V-FITC and propidium iodide (PI) (n=3 biologically independent animals per group). Quantification showed that deletion of *Txndc5* did not increase the percentage of apoptosis lung

fibroblasts (Q2+Q4). (n.s.: non-significant determined using two-tailed Mann-Whitney *U* test. Source data are provided as a Source Data file. MLF: mouse lung fibroblasts).

Fig. 1b

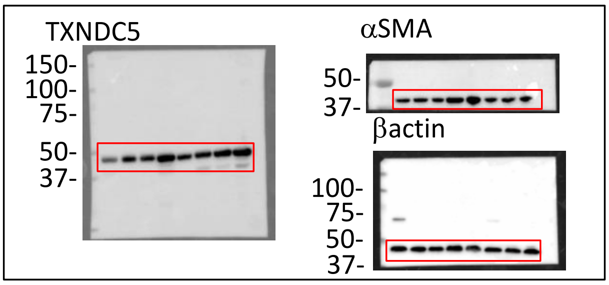

Fig. 1c

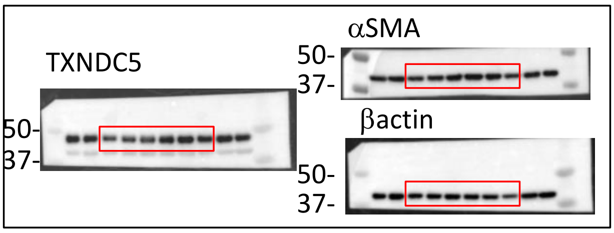

Fig. 5a

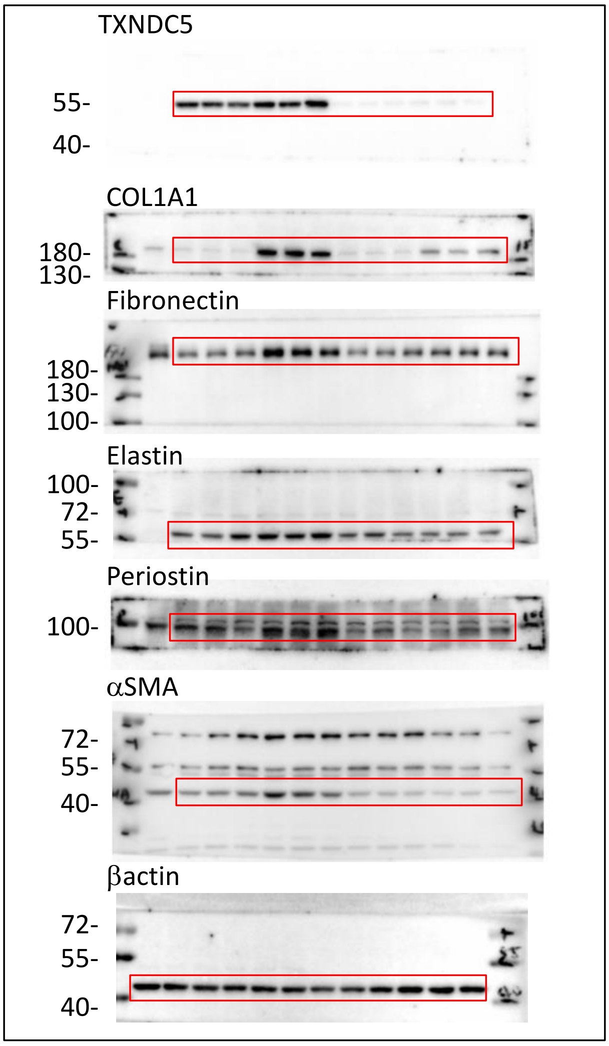

Fig. 5d

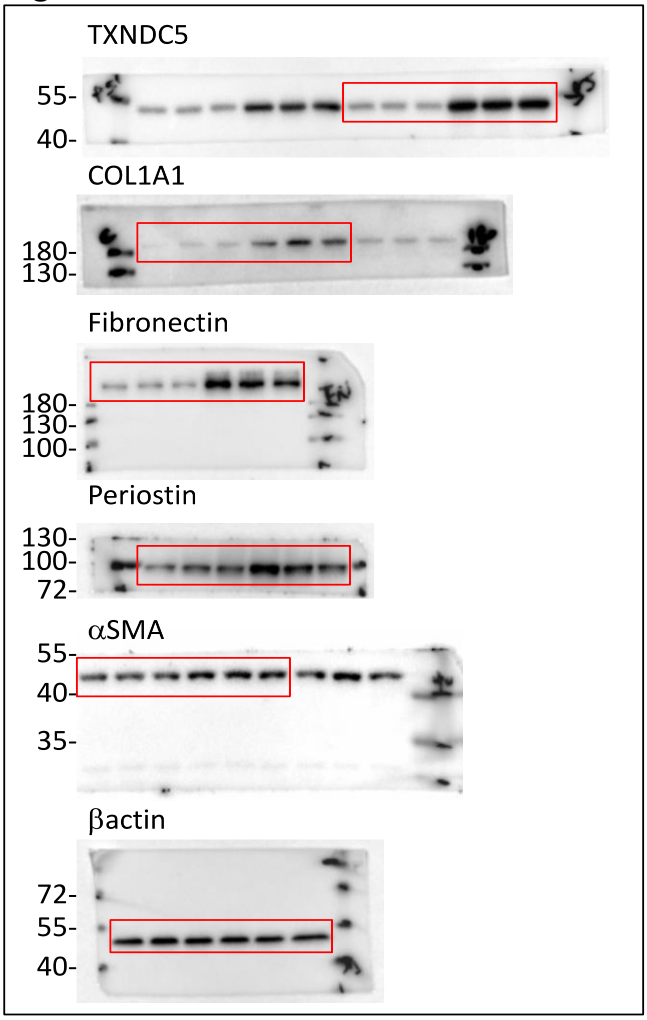

Fig. 6a

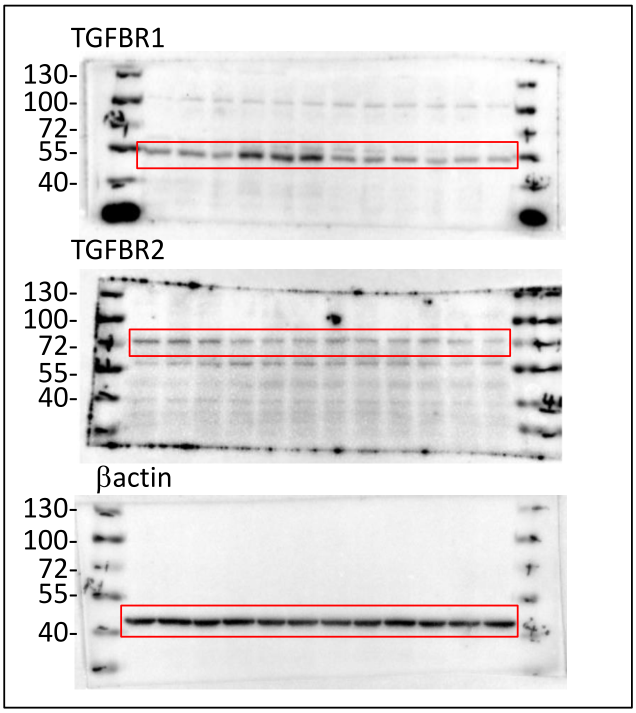

Fig. 6b

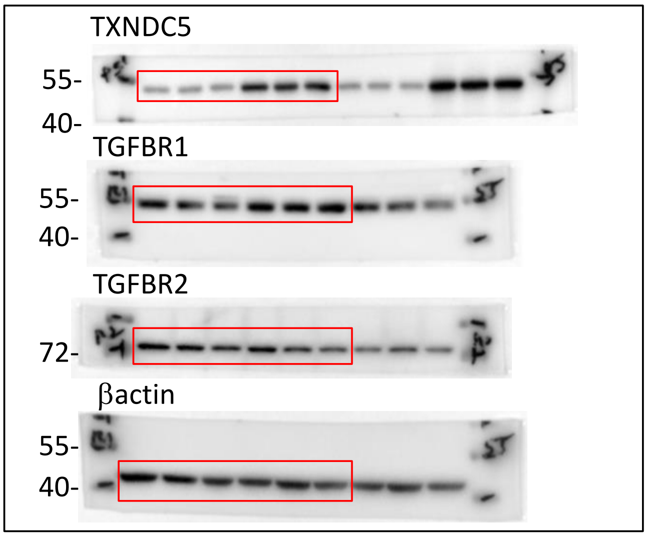

Fig. 6c

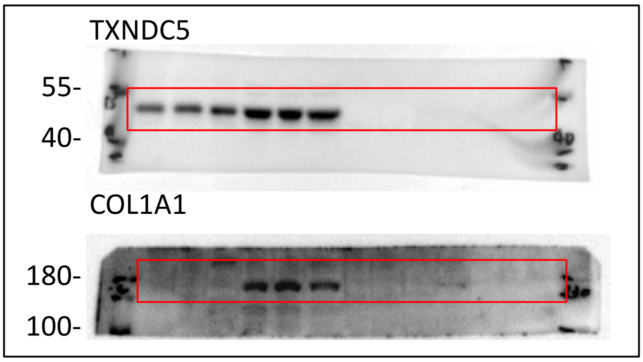

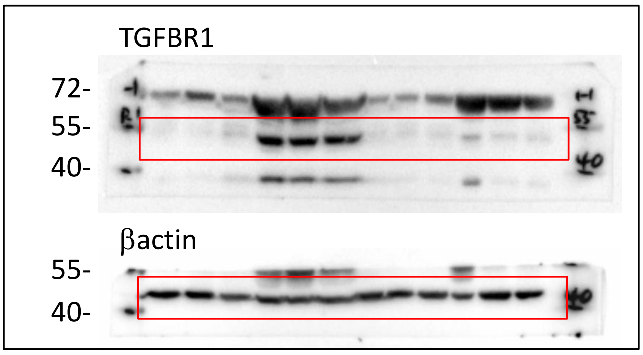

Fig. 7a

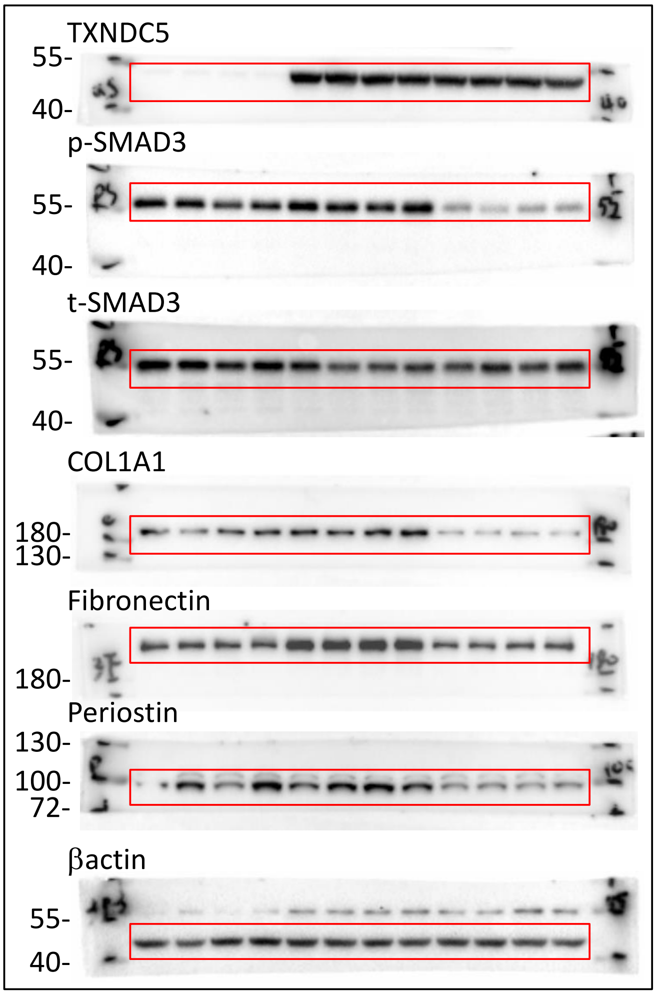

Fig. 7b

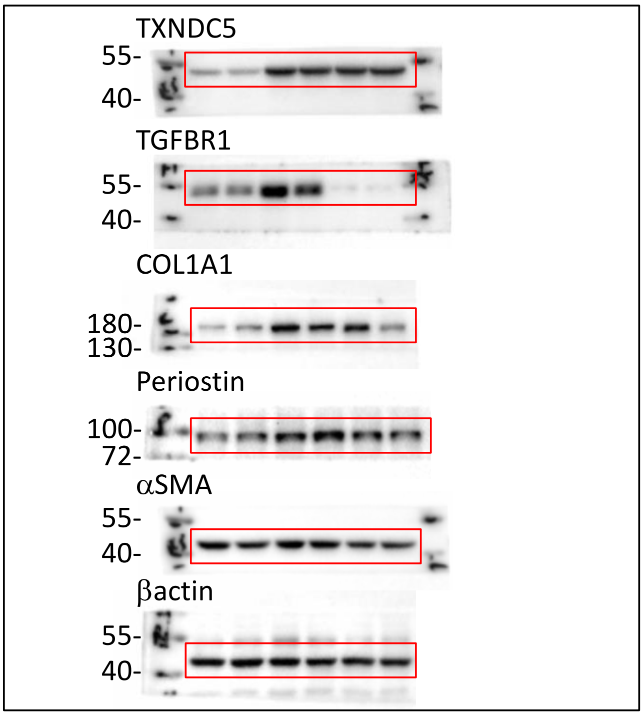

Fig. 8a

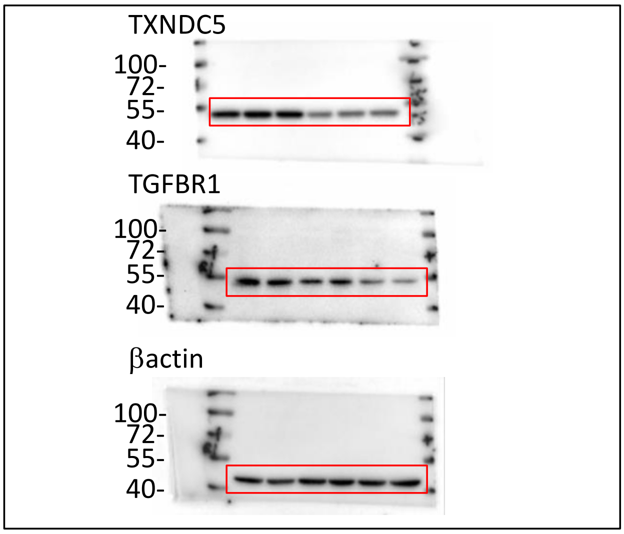

Fig. 8b

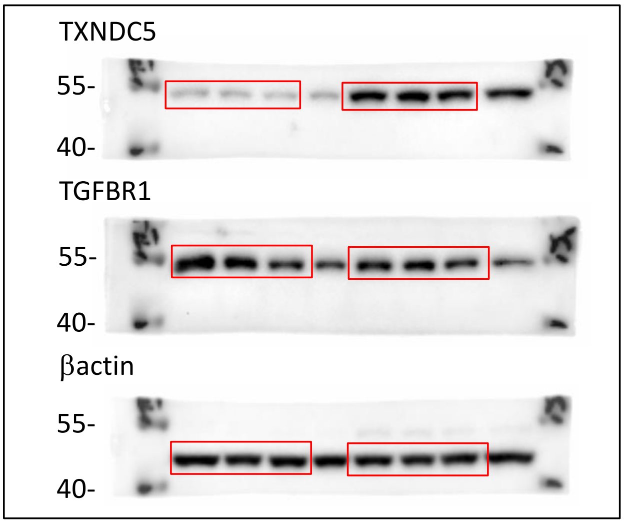

Fig. 8c

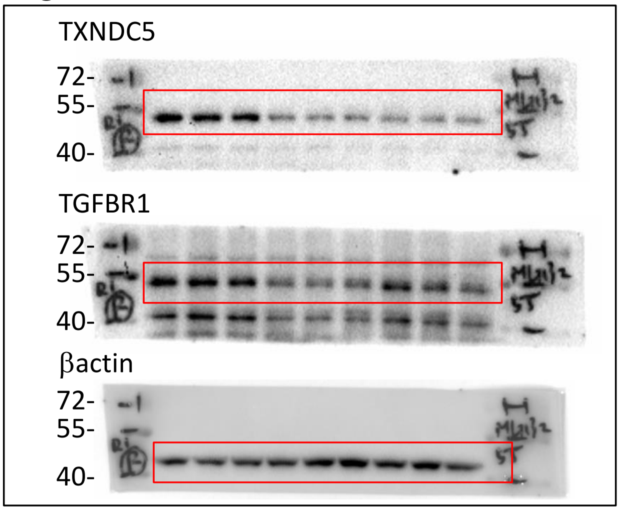

Fig. 8d

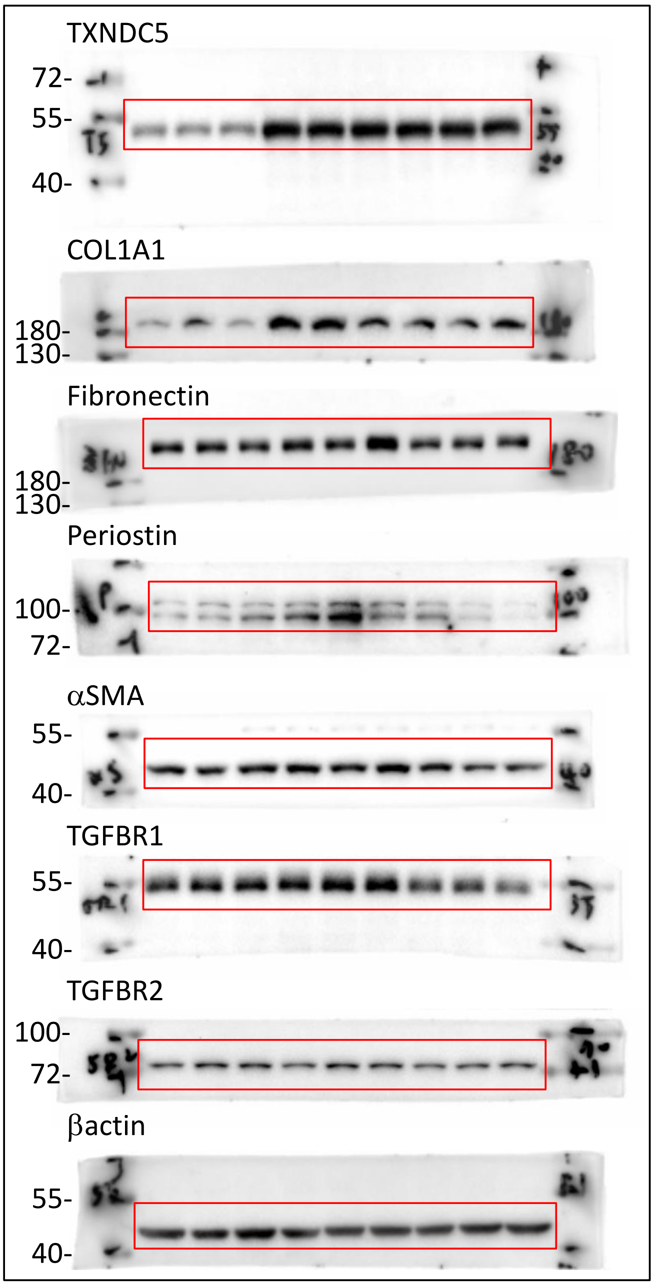

Fig. 9a

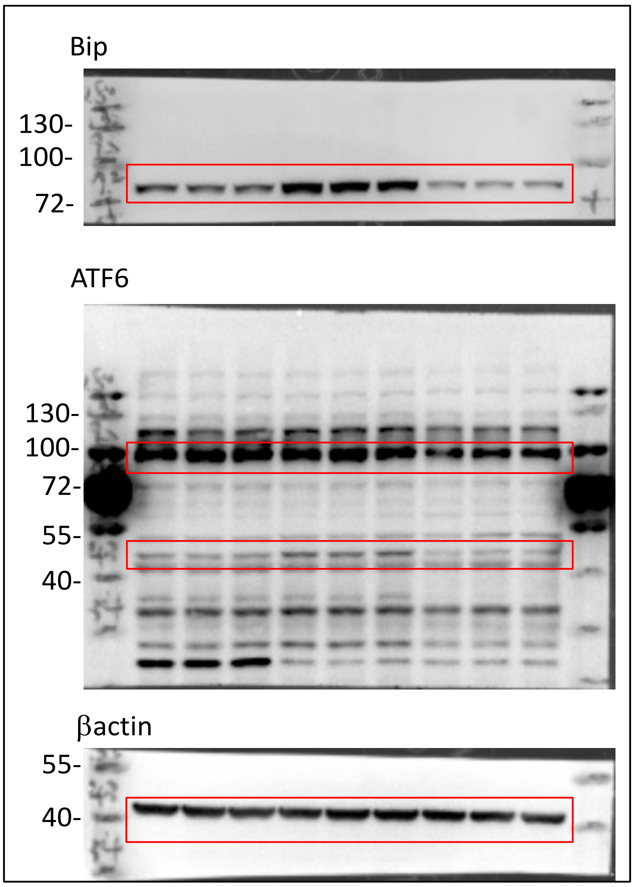

Supplementary Fig. 6a

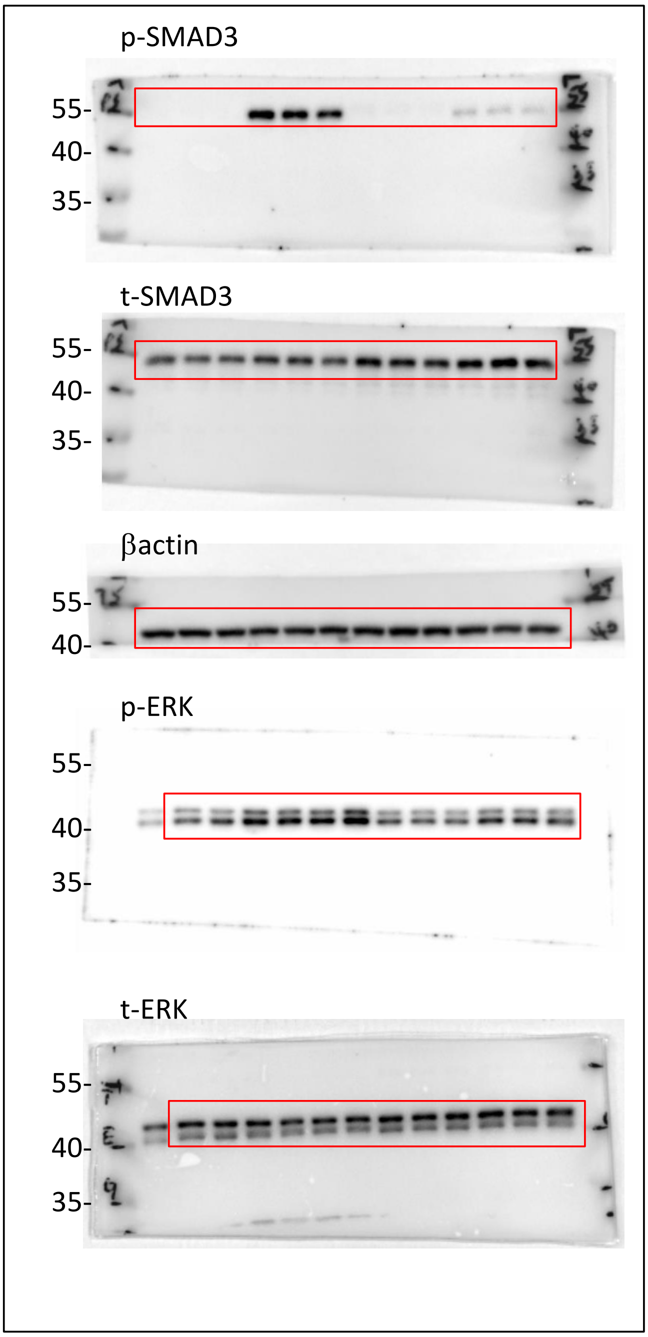

Supplementary Fig. 5a

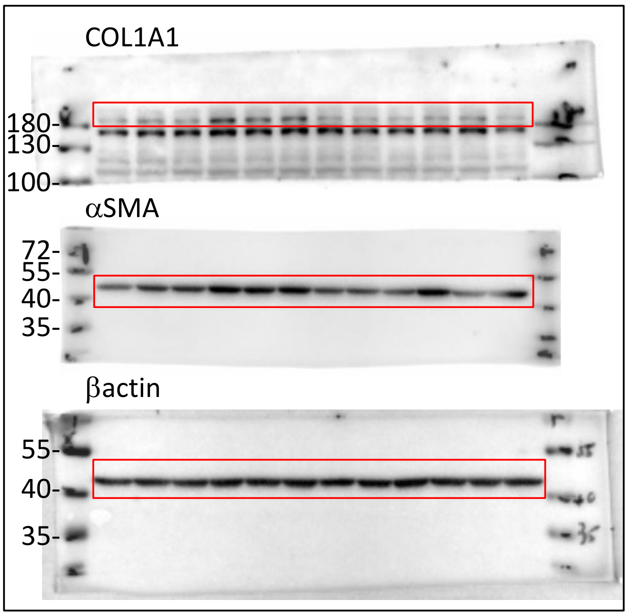

Supplementary Fig. 6a

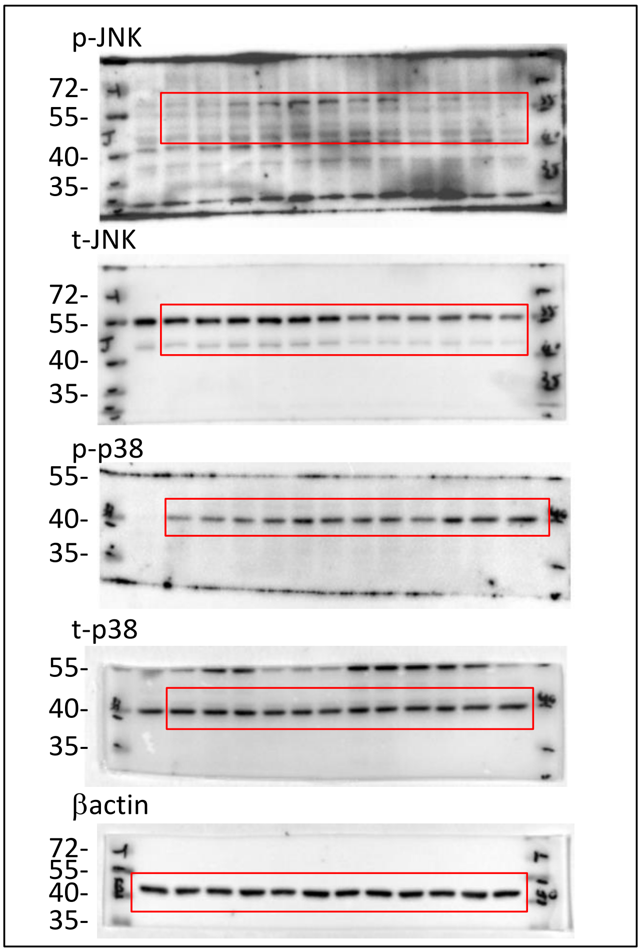

Supplementary Fig. 6d

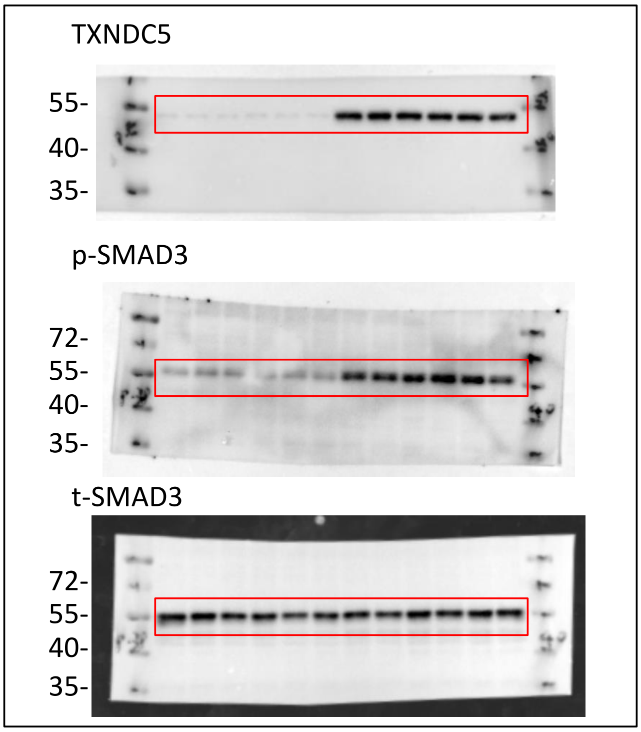

Supplementary Fig. 6d

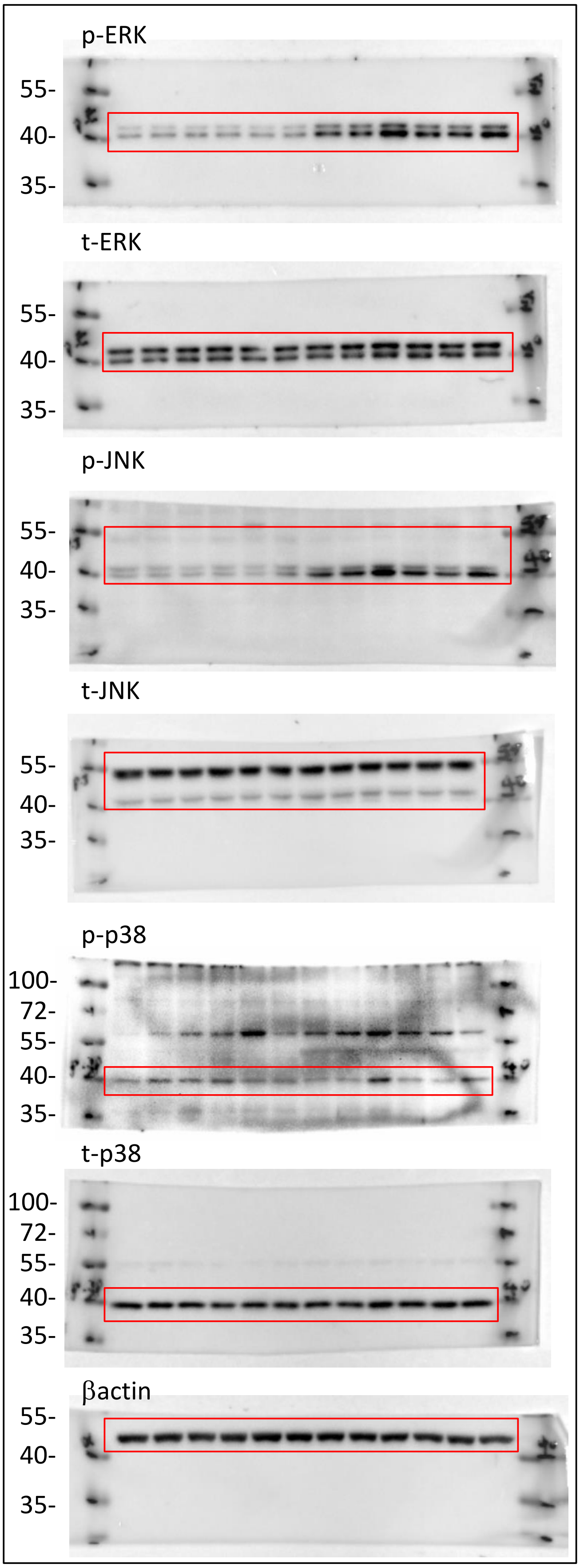

Supplementary Fig. 8c

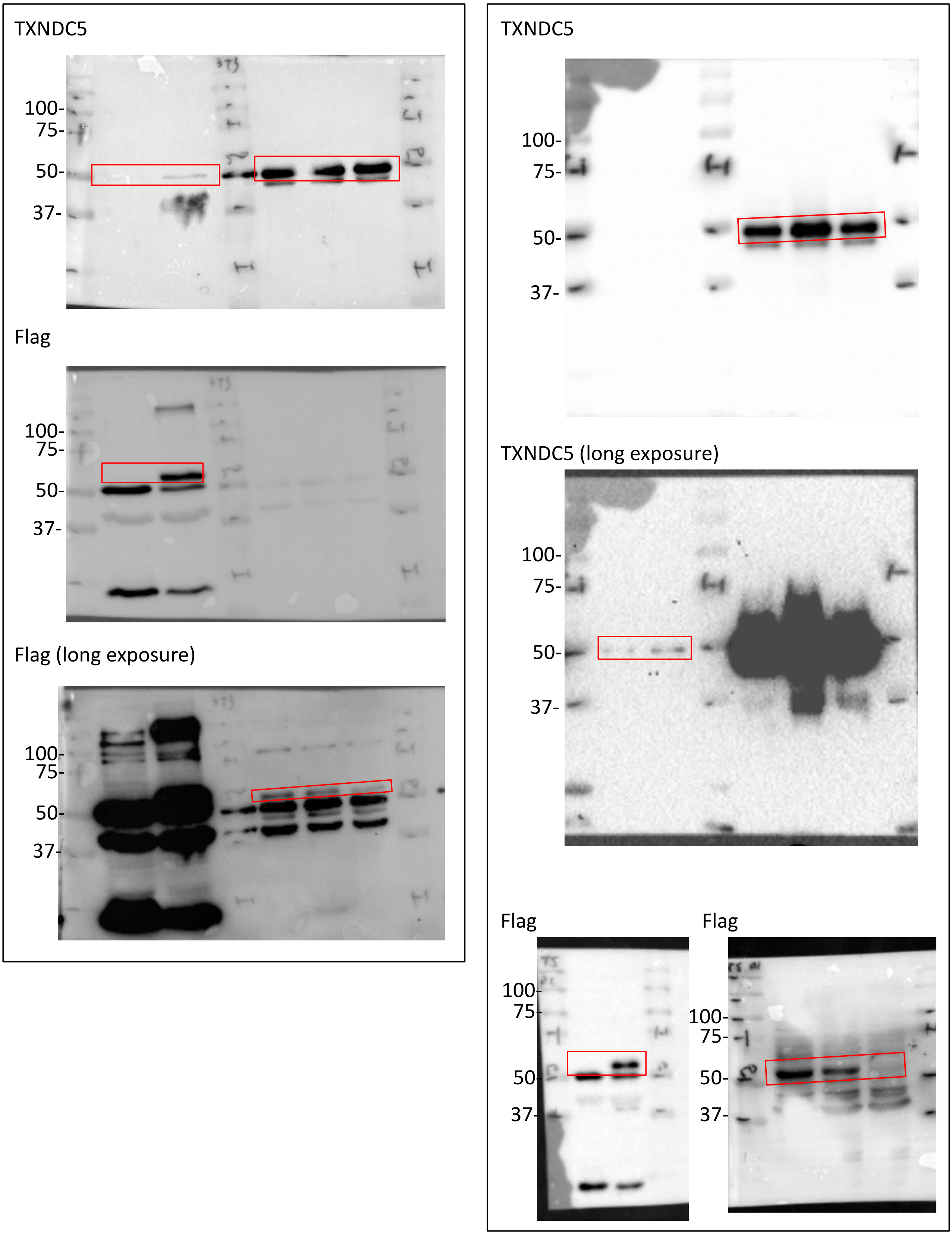

Supplementary Fig. 8d

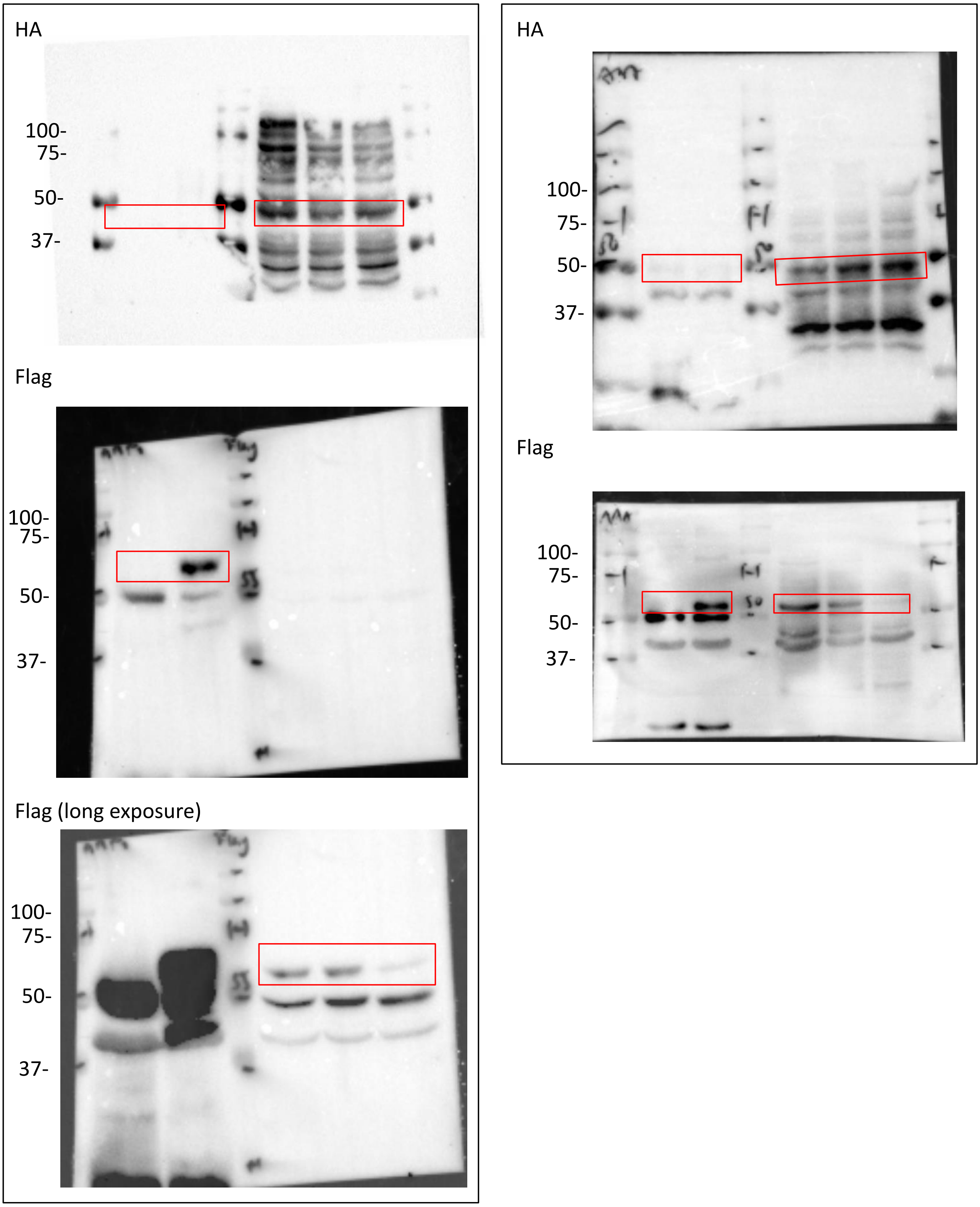

Supplementary Fig. 9a

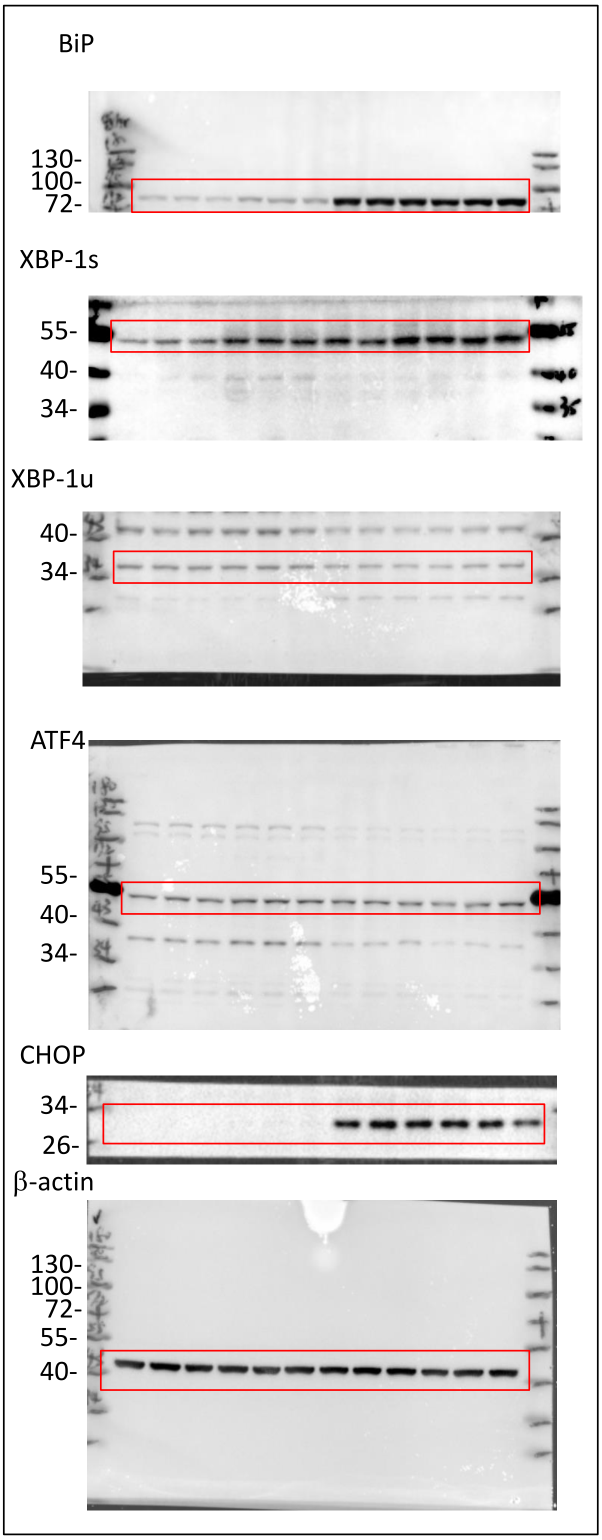

Supplementary Fig. 14a

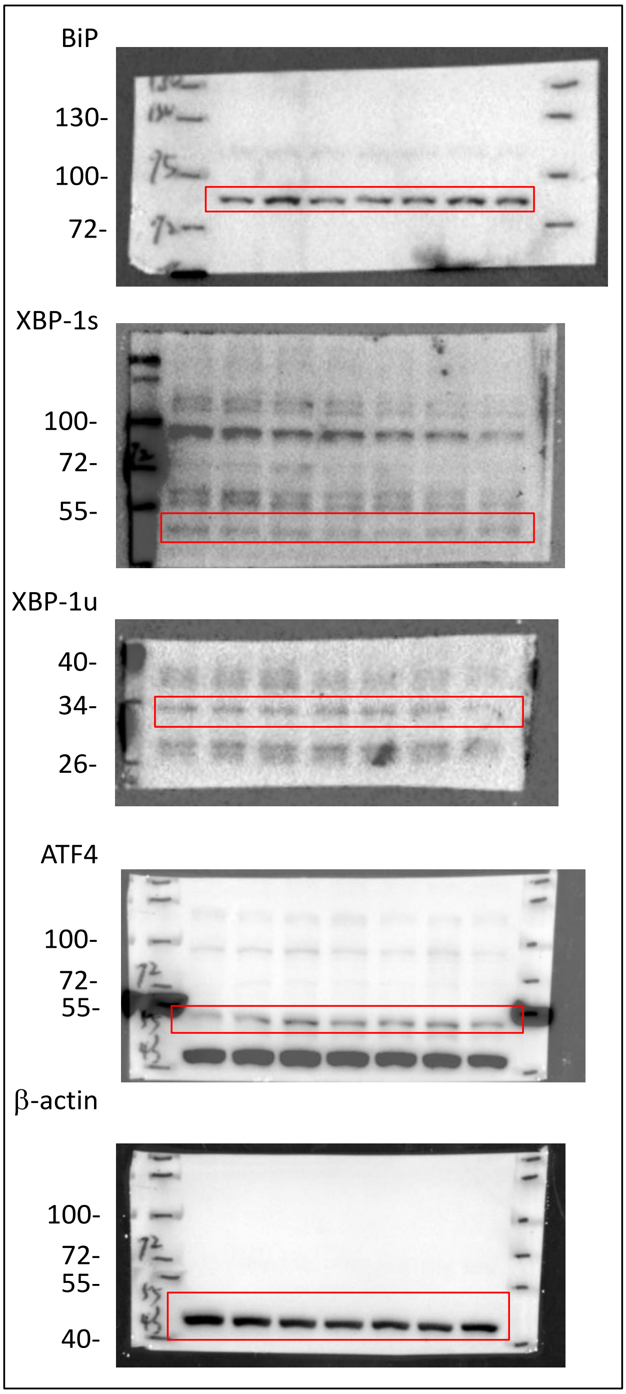

Supplementary Fig. 14c

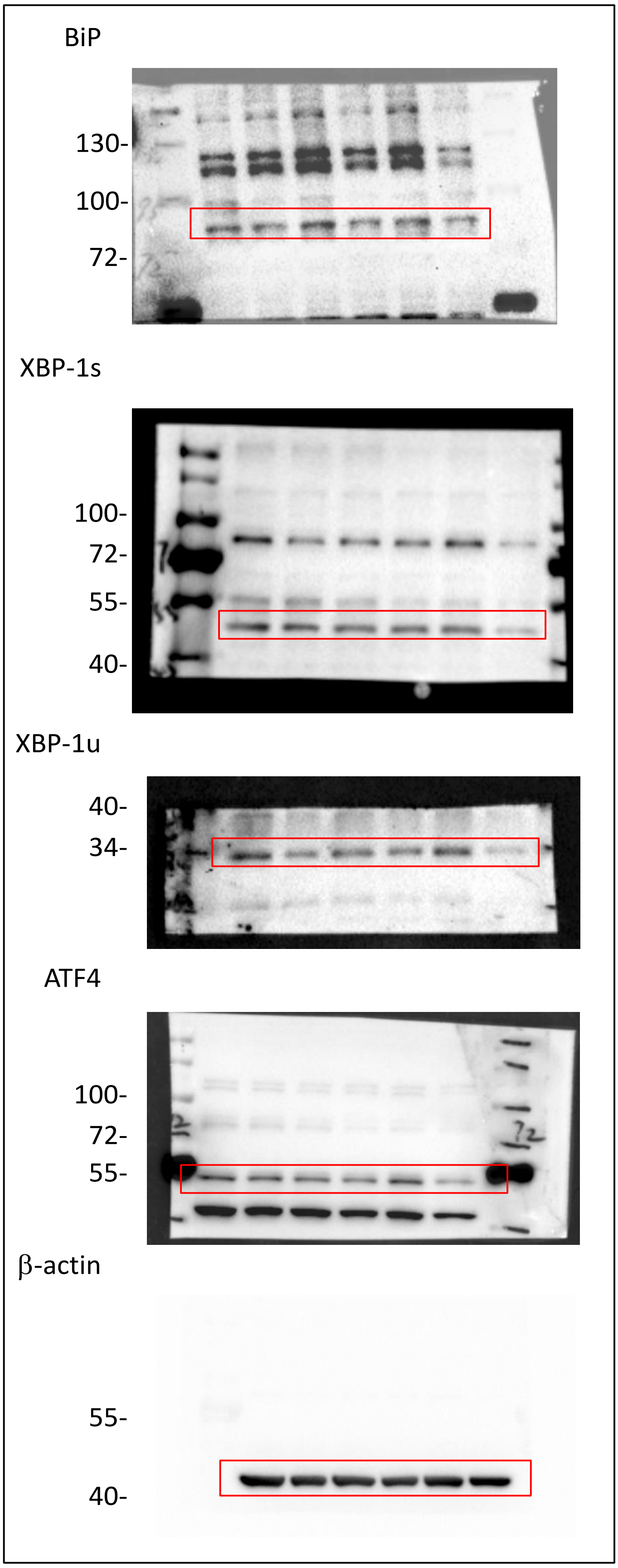

Supplementary Fig. 15 Original Western blots

Raw data in Supplementary Fig. 14

WT MLF

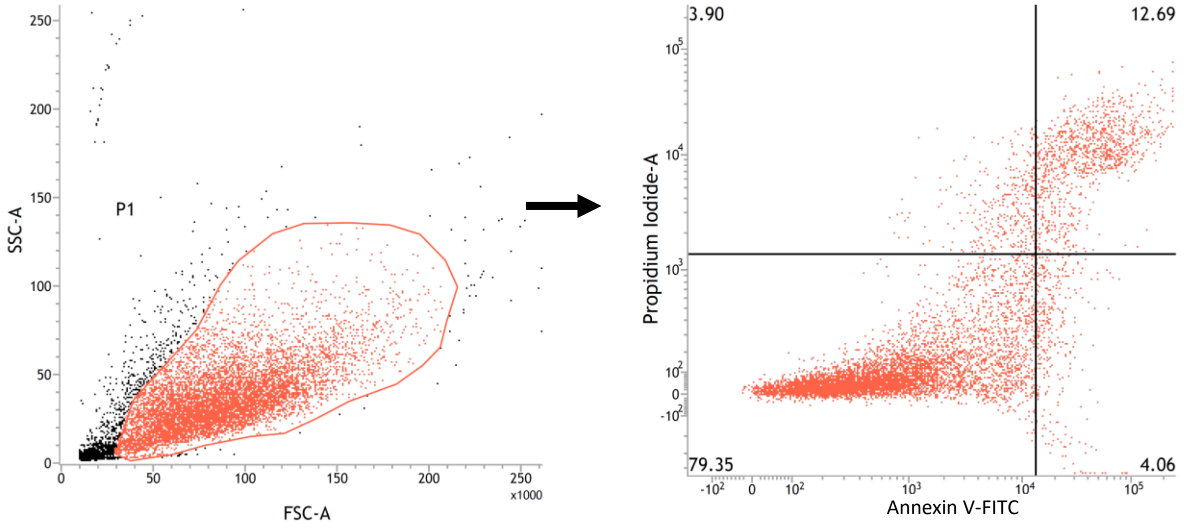

Txndc5$^{-/-}$ MLF

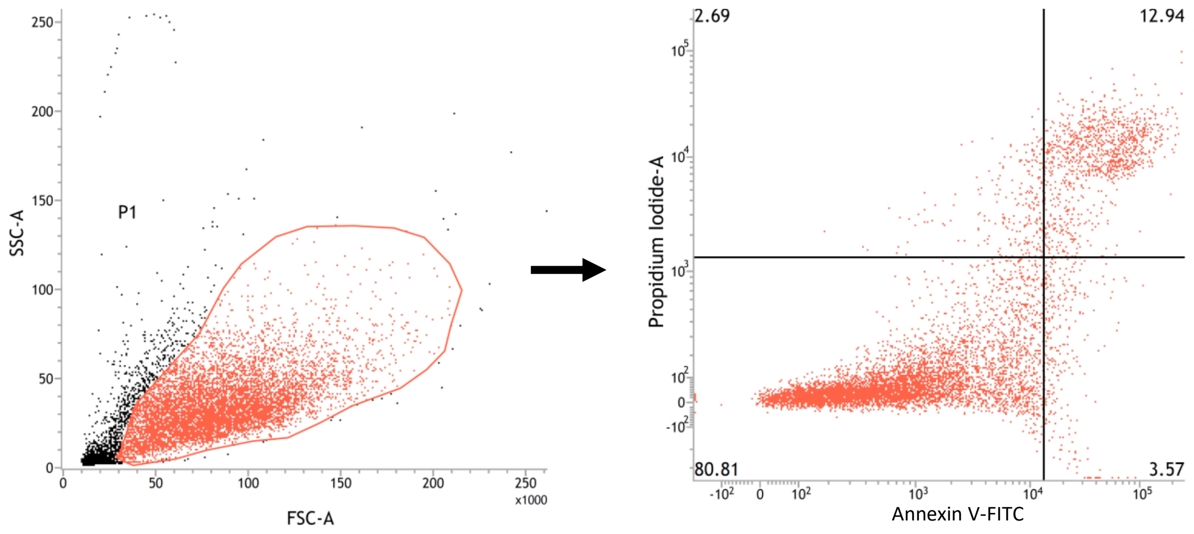

Supplementary Fig. 16 Gating strategies for supplementary fig. 14e

Supplementary Table 1 Primer sequences used in this study.

a

Gene	Mouse primer Sequence	
Acta2	F:5'GAGCTACGAACTGCCTGACG3'	R:5'TACCCCCTGACAGGACGTTG3'
Col1a1	F: 5'TTCTCCTGGCAAAGACGGAC3'	R: 5'CGGCCACCATCTTGAGACTT3'
Col3a1	F: 5'TCACCAGGACAAAGAGGGGA3'	R: 5'CCACCAGGACTGCCGTTATT3'
Ctgf	F: 5'GTGCACTGCCAAGATGGTG3'	R: 5'CTTTGGAAGGACTCACCGCT3'
Eln	F: 5'GTATGGCCTTGGTGGAGCC3'	R: 5'AAGGCCAGCAGCACCATATT3'
Fn	F: 5'ACAAACACTAATGTTAATTGCCCA3'	R: 5'CGGGAATCTTCTCTGTCAGCC3'
Hprt	F:5'GTTGGGCTTACCTCACTGCT3'	R:5'TAATCACGACGCTGGGACTG3'
Il1b	F: 5'TGCCACCTTTTGACAGTGATG3'	R: 5'ATGTGCTGCTGCGAGATTTG3'
Il6	F: 5'TCAATATTAGAGTCTCAACCCCCA3'	R: 5'GAAGGCGCTTGTGGAGAAGG3'
Txndc5	F:5'GAAAGCGGGACTTGGAGTCA3'	R:5'AGTCCACTTCTGCGATGGTG3'

b

Gene	Human primer Sequence	
ACTA2	F: 5'CTCCCAGGGCTGTTTTCCCA3'	R: 5'CCATGTCGTCCCAGTTGGTG3'
ATF6	F: 5'TCATGTCTATGAACCCATCCTCG 3'	R: 5'ACCTCCTTGTCAGCCCCTAAT3'
COL1A1	F: 5'CACAGAGGTTTCAGTGGTTTGG3'	R:5'AGTAGCACCATCATTTCCACGA3'
FN	F:5'ACAAACACTAATGTTAATTGCCCA3'	R:5'CGGGAATCTTCTCTGTCAGCC3'
ELN	F:5'CAGTTGGTACCCAAGCACCT3'	R:5'AGGTGGCTATTCCCAGTGTG3'
HPRT	F:5'CGTCTTGCTCGAGATGTGATG3'	R:5'GCACACAGAGGGCTACAATGTG3'
TXNDC5	F:5'CGTGGTCTTCGAGAAATGCG3'	R:5'AGGTCATTCCAAGTCGGCTG3'
XBP1	F:5'CGAGGAGAAGGCGCTGAG	R:5' CAGCTCACTCATTCGAGCCT
EIF2A	F:5'CCCAACCATACAAGGTGGCT3'	R:5'TGCATGAGGTCCAGCAAAGT3'

Reference

1. Lindahl, G.E., *et al.* Microarray profiling reveals suppressed interferon stimulated gene program in fibroblasts from scleroderma-associated interstitial lung disease. *Respir Res* **14**, 80 (2013).
