# Supplementary material for: Fibroblast-enriched endoplasmic reticulum protein TXNDC5 promotes pulmonary fibrosis by augmenting TGFβ signaling through TGFBR1 stabilization
Source: Nat Commun. 2020 Aug 26;11:4254. doi: 10.1038/s41467-020-18047-x (PMC7449970; doi:10.1038/s41467-020-18047-x)
Supplement: Supplementary file 3 — Reporting Summary [file 41467_2020_18047_MOESM3_ESM.pdf]

nature research

Corresponding author(s): Kai-Chien Yang, MD, PhD

Last updated by author(s): Jul 9, 2020

Reporting Summary

Nature Research wishes to improve the reproducibility of the work that we publish. This form provides structure for consistency and transparency in reporting. For further information on Nature Research policies, see Authors & Referees and the Editorial Policy Checklist .

Statistics

For all statistical analyses, confirm that the following items are present in the figure legend, table legend, main text, or Methods section.

n/a	Confirmed	
☐	☒	The exact sample size (*n*) for each experimental group/condition, given as a discrete number and unit of measurement
☐	☒	A statement on whether measurements were taken from distinct samples or whether the same sample was measured repeatedly
☐	☒	The statistical test(s) used AND whether they are one- or two-sided<br>*Only common tests should be described solely by name; describe more complex techniques in the Methods section.*
☐	☒	A description of all covariates tested
☐	☒	A description of any assumptions or corrections, such as tests of normality and adjustment for multiple comparisons
☐	☒	A full description of the statistical parameters including central tendency (e.g. means) or other basic estimates (e.g. regression coefficient) AND variation (e.g. standard deviation) or associated estimates of uncertainty (e.g. confidence intervals)
☐	☒	For null hypothesis testing, the test statistic (e.g. *F*, *t*, *r*) with confidence intervals, effect sizes, degrees of freedom and *P* value noted<br>*Give P values as exact values whenever suitable.*
☒	☐	For Bayesian analysis, information on the choice of priors and Markov chain Monte Carlo settings
☒	☐	For hierarchical and complex designs, identification of the appropriate level for tests and full reporting of outcomes
☒	☐	Estimates of effect sizes (e.g. Cohen's *d*, Pearson's *r*), indicating how they were calculated

Our web collection on statistics for biologists contains articles on many of the points above.

Software and code

Policy information about availability of computer code

Data collection	ImageLab software version 5.1, GPU-Nrecon Software, SkyScan 2211, flexiWare, cellSens Standard 1.14 software, Gen5, BD FACSuite software
Data analysis	GraphPad Prism 6.0, ImageJ, CTAn software (1.16.4.1), Instarecon, BD FACSuite software

For manuscripts utilizing custom algorithms or software that are central to the research but not yet described in published literature, software must be made available to editors/reviewers. We strongly encourage code deposition in a community repository (e.g. GitHub). See the Nature Research guidelines for submitting code & software for further information.

Data

Policy information about availability of data

All manuscripts must include a data availability statement. This statement should provide the following information, where applicable:

- Accession codes, unique identifiers, or web links for publicly available datasets
- A list of figures that have associated raw data
- A description of any restrictions on data availability

The data that support the findings of this study are available from the corresponding author upon reasonable request. The source data underlying Figs 1-10 and Supplementary Figs 1-10,12-14 are provided as a Source Data file.

Field-specific reporting

Please select the one below that is the best fit for your research. If you are not sure, read the appropriate sections before making your selection.

Life sciences study design

All studies must disclose on these points even when the disclosure is negative.

Sample size	Power analyses were conducted on the results of pilot experiments to determine the sample size required to obtain statistically significant results. Total sample sizes (n) are provided for each of the experiments required statistical analysis in the figure legends.
Data exclusions	No data were excluded.
Replication	All data (except for supplementary figures 2c-g and 8c-8d) shown were obtained from at least 3 biological independent experiments. Supplementary figures 2c-g and 8c-8d were performed by 2 biological independent experiments.
Randomization	A block randomization method was used to assign experimental animals to groups on a rolling basis to achieve adequate sample number for each experimental condition. Other samples were allocated random.
Blinding	All experimental animals were assigned unique identifiers to blind experimenters to genotypes and treatment. For other experiments, analysts were blinded.

Reporting for specific materials, systems and methods

We require information from authors about some types of materials, experimental systems and methods used in many studies. Here, indicate whether each material, system or method listed is relevant to your study. If you are not sure if a list item applies to your research, read the appropriate section before selecting a response.

Materials & experimental systems

n/a	Involved in the study	
☐	☒	Antibodies
☐	☒	Eukaryotic cell lines
☒	☐	Palaeontology
☐	☒	Animals and other organisms
☐	☒	Human research participants
☒	☐	Clinical data

Methods

n/a	Involved in the study	
☒	☐	ChIP-seq
☐	☒	Flow cytometry
☒	☐	MRI-based neuroimaging

Antibodies

Antibodies used

TXNDC5 (Proteintech, IL, USA, 19834-1-AP)
TGFBR1 (OriGene, MD, USA, AP14647PU-N)
SMA (Sigma Aldrich, MO, USA, A5228)
COL1A1 (EMD Millipore, CA, USA, AB765P, for mouse species)
COL1A1 (1:1000, OriGene, MD, USA, TA309096, for human species)
FN (1:1000, BD Biosciences, CA, USA, 610077)
ELN, POSTN, XBP-1u, ATF4(GeneTex, CA, USA, GTX37428, GTX100602, GTX113295, GTX101943)
total/p-JNK, total/p-ERK, total/p-38, BiP, XBP-1s, CHOP (Cell Signaling Technology, MA, USA, 9252, 9251, 9102, 4370, 9212, 9211, 3177, 12782, 2895)
SMA, total/p-SMAD3, HA-tag (Abcam, Cambridge, UK, ab5694, ab52903, ab40854, ab9110)
TGFBR1 (Thermo Fisher Scientific, MA, USA, PA5-32631, for human species)
TGFBR1 (Abcam, Cambridge, UK, ab31013, for mouse species)
TGFBR2 (OriGene, MD, USA, TA311643)
ATF6 (Bio Academia, Osaka, Japan, 73-505)
Flag-tag (Cell Signaling Technology, MA,USA, 8146)
beta-actin (OriGene, MD, USA, TA811000)
Alexa Fluor 594-labeled anti-rabbit secondary antibody (BioLegend, 406418)
Alexa Fluor 488-labeled anti-mouse secondary antibody (BioLegend, 405319)

Validation

All the antibodies were from commercial sources and have been validated by the manufacturers. The validation data are available on the vendor's website.
TXNDC5 antibody (Proteintech, IL, USA, 19834-1-AP) was further validated by Txndc5 KO mouse lung using immunoblotting and the results were shown in Fig. 6c.
TGFBR1 (OriGene, MD, USA, AP14647PU-N) was validated by human cancer tissue using IHC.
SMA (Sigma Aldrich, MO, USA, A5228) was validated by human appendix sections using IF.
COL1A1 (EMD Millipore, CA, USA, AB765P) was validated by mouse skin sections using IHC and by mouse liver lysate using immunoblotting.
COL1A1 (OriGene, MD, USA, TA309096) was validated by rat lung lysate using immunoblotting.

FN (BD Biosciences, CA, USA, 610077) was validated by A-431 cell lysate using immunoblotting.
ELN (GeneTex, CA, USA, GTX37428) was validated by mouse stomach lysate using immunoblotting.
POSTN, ATF4 (GeneTex, CA, USA, GTX100602, GTX101943), total-ERK, XBP-1s (Cell Signaling Technology, MA, USA, 9102, 12782), SMA (Abcam, Cambridge, UK, ab5694) were validated by HeLa cell lysate using immunoblotting.
XBP-1u (GeneTex, CA, USA, GTX113295), total/p-JNK, Flag-tag (Cell Signaling Technology, MA, USA, 9252, 9251, 8146), HA-tag (Abcam, Cambridge, UK, ab9110) were validated by 293T lysate using immunoblotting.
p-ERK (Cell Signaling Technology, MA, USA, 4370) ,total/p-38 (Cell Signaling Technology, MA, USA, 9212, 9211), TGFBR2 (OriGene, MD, USA, TA311643), ATF6 (Bio Academia, Osaka, Japan, 73-505) were validated by NIH/3T3 lysate using immunoblotting.
BiP, CHOP (Cell Signaling Technology, MA, USA, 3177, 2895) were validated by A024 lysate using immunoblotting.
total-SMAD3 (Abcam, Cambridge, UK, ab52903), TGFBR1(Thermo Fisher Scientific, MA, USA, PA5-32631), beta-actin (Origene, MD, USA, TA811000) were validated by A549 lysate using immunoblotting.
p-SMAD3 (Abcam, Cambridge, UK, ab40854) was validated by HT-29 lysate using immunoblotting.
TGFBR1 (Abcam, Cambridge, UK, ab31013) was validated by mouse intestine whole tissue lysate using immunoblotting.

Eukaryotic cell lines

Policy information about cell lines

Cell line source(s)	Human Pulmonary Fibroblasts-adult (ScienCell #3310), NIH/3T3 (ATCC® CRL-1658™), isolated primary mouse lung fibroblasts
Authentication	None of the cell lines used were authenticated.
Mycoplasma contamination	All cell lines were not tested for mycoplasma infection.
Commonly misidentified lines (See ICLAC register)	No commonly misidentified cell lines were used.

Animals and other organisms

Policy information about studies involving animals; ARRIVE guidelines recommended for reporting animal research

Laboratory animals	Male mice aging between 8-10 weeks were used.<br>C57BL/6, Txndc5-/-, Col1a2-cre/ERT2, Txndc5fl/fl, Tie2-Cre/ERT2*ROSA26-tdTomato, SPC-Cre/ERT2*ROSA26-mTmG, Col1a1-GFP reporter mice.<br>All mice were on a C57Bl/6 background. Experimental procedures were approved by the relevant animal experimentation. committee and conducted in compliance with international and local animal welfare legislations.
Wild animals	This study did not involve wild animals.
Field-collected samples	This study did not involve samples collected from the field.
Ethics oversight	The animal work was approved by the institutional review boards of National Taiwan University and University of Chicago

Note that full information on the approval of the study protocol must also be provided in the manuscript.

Human research participants

Policy information about studies involving human research participants

Population characteristics	This study is composed of explanted lungs from 13 IPF patients obtained at the time of lung transplantation (2 female, 10 male and 1 sex not available) with average age (mean± SEM): 62±3.0 years old, fibrosis score 1/2/3 (<25% = 1, 25-75% = 2, >75% = 3): 0/2/11, and lungs unsuitable for transplantation from 9 non-IPF donor patients (3 female and 6 male) with average age (mean± SEM): 49±3.9 years old, fibrosis score 1/2/3 (<25% = 1, 25-75% = 2, >75% = 3): 8/1/0
Recruitment	Non-IPF control human lung samples were obtained from de-identified human lungs declined for transplantation (whose lungs were declined for transplantation, but whose next of kin had given consent for use in research) through the Regional Organ Bank of Illinois (ROBI) and Gift of Hope in collaboration with Dr. Julian Solway and Dr. Ann Sperling at the University of Chicago. IPF lung samples were obtained from de-identified explanted lungs of IPF patients undergoing lung transplantation at the University of Chicago. A written informed consent was obtained for each of the IPF patients. No self-selection bias.
Ethics oversight	All studies involving human lung tissues were approved by the University of Chicago IRB.

Note that full information on the approval of the study protocol must also be provided in the manuscript.

Flow Cytometry

Plots

Confirm that:

- [x] The axis labels state the marker and fluorochrome used (e.g. CD4-FITC).
- [x] The axis scales are clearly visible. Include numbers along axes only for bottom left plot of group (a 'group' is an analysis of identical markers).
- [x] All plots are contour plots with outliers or pseudocolor plots.
- [x] A numerical value for number of cells or percentage (with statistics) is provided.

Methodology

Sample preparation	Murine primary lung fibroblasts isolation was prepared as described in methods section.
Instrument	BD FACSCaliburTM flow cytometer
Software	BD FACSuite software
Cell population abundance	Analysis 8*10^3 cells with post-sort populations. Cell were gated by FSC and SSC to discard cell adhesion and debris.
Gating strategy	All gating strategies are provided in 'Source data'

- [x] Tick this box to confirm that a figure exemplifying the gating strategy is provided in the Supplementary Information.
